# All‐3D‐Printed Multi‐Environment Modular Microrobots Powered by Large‐Displacement Dielectric Elastomer Microactuators

**DOI:** 10.1002/adma.202507503

**Published:** 2025-09-09

**Authors:** Won Jun Song, Yong‐Woo Kang, Yun Hyeok Lee, Junhyung Kim, Bastien F.G. Aymon, Seong‐Yu Choi, Yong Eun Cho, Xiao‐Yun Yan, Shucong Li, Younghoon Lee, Xuanhe Zhao, Yong‐Lae Park, Jeong‐Yun Sun

**Affiliations:** ^1^ Departmant of Materials Science and Engineering Seoul National University Seoul 08826 Republic of Korea; ^2^ Department of Mechanical Engineering Seoul National University Seoul 08826 Republic of Korea; ^3^ Institute of Engineering Research Seoul National University Seoul 08826 Republic of Korea; ^4^ Department of Mechanical Engineering Massachusetts Institute of Technology Cambridge MA 02139 USA; ^5^ Department of Mechanical Engineering Kyung Hee University Yongin 17104 Republic of Korea; ^6^ Research Institute of Advanced Materials Seoul National University Seoul 08826 Republic of Korea; ^7^ Institute of Advanced Machines and Design Seoul National University Seoul 08826 Republic of Korea

**Keywords:** dielectric elastomer actuators, microrobots, modular design, multimaterial 3D printing

## Abstract

Microrobots are expected to push the boundaries of robotics by enabling navigation in confined and cluttered environments due to their sub‐centimeter scale. However, most microrobots perform best only in the specific conditions for which they are designed and require complete redesign and fabrication to adapt to new tasks and environments. Here, fully 3D‐printed modular microrobots capable of performing a broad range of tasks across diverse environments are introduced. For multi‐environment navigation, large‐displacement dielectric elastomer microactuators with a soft‐stiff hybrid structure are developed, capable of powering microrobots to stride over obstacles on various terrestrial terrain and rapidly propel themselves across aquatic terrain. To further expand their capabilities beyond mere navigation, ten task‐specific modules for the microrobots are developed. All modules are fabricated using a digital light processing multimaterial 3D printer capable of simultaneously printing multiple photocurable resins, providing a broadly applicable platform for fabricating mesoscale robotic components. The microrobots navigate across smooth, rough, granular, and aquatic environments, demonstrating tasks such as controlling the movements of nearby robots, interacting with humans to avoid collisions, and collaboratively dragging heavy objects through multi‐unit operation. The study addresses key limitations hindering the integration of modular design into microrobots, enabling adaptation to new environments and tasks.

## Graphical abstract

Fully 3D‐printed modular microrobots capable of performing a broad range of tasks across diverse environments are demonstrated. The key breakthrough lies in the development of a large‐displacement microactuator for multi‐environment navigation and a mesoscale multimaterial 3D printing platform for on‐demand module fabrication. Together, these innovations advance the adaptability, versatility, and scalability of microrobotic systems.

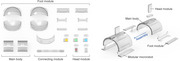

## 1 Introduction

Microrobots, with their sub‐centimeter size, hold vast potential for applications such as reconnaissance, exploration, and rescue missions that require operation in confined and cluttered environments.^[^ ^1^ ^]^ However, designing microrobots presents distinct challenges that differ from those of macroscale robots due to the inefficiencies arising from scaling laws.^[^ ^2^ ^]^ Moreover, fabricating components within the micrometer‐to‐millimeter range requires specialized mesoscale manufacturing techniques.^[^ ^1^, ^2^, ^3^ ^]^ Consequently, microrobot research has focused on optimizing designs tailored to particular environments and tasks, along with their fabrication processes. While effective, this approach often limits versatility, as most microrobots perform best only in the specific conditions for which they were originally designed. This necessitates costly and time‐consuming redesign and fabrication to adapt to new tasks or environments.

Developing microrobots capable of performing diverse tasks across various environments requires strategies that enable structural and functional modifications with minimal cost and effort. A modular design, which subdivides a system into smaller, function‐specific components—each independently designed, fabricated, and replaced—offers a promising solution, as it is expected to allow microrobots to adapt to various tasks and environments without needing a complete redesign and fabrication.^[^ ^4^ ^]^ However, applying modular design to microrobots presents two major challenges. The first is the need for microactuators that meet the diverse requirements for navigation in various unstructured environments where microrobots are expected to be deployed, including large deformation, broad bandwidth, high power density, and reliability. The second is the need for a broad range of mesoscale components—such as sensors, structural elements, and communication units—that are essential for enabling microrobots to perform tasks beyond mere navigation. These two challenges have thus far limited the integration of modular design into microrobotic systems.

Among the various actuation technologies, dielectric elastomer actuators (DEAs) are recognized for their high power density, fast response speed, and physical robustness against external impacts, making them widely used in fields such as soft robotics and microrobotics.^[^ ^3^, ^5^ ^]^ At the macroscale, DEAs, typically ranging from a few centimeters and larger, incorporate stiff elements like films, fibers, and frames to convert small in‐plane actuation into larger out‐of‐plane deformations.^[^ ^6^ ^]^ However, for dielectric elastomer microactuators (mDEAs) used in microrobots, the constraints of mesoscale multimaterial fabrication—from soft to rigid and from conductive to dielectric—pose challenges in applying such soft–stiff hybrid designs. As a result, mDEAs are composed entirely of soft materials, generating limited linear displacement (|Δ*L*_f_|/*L*_0_ < 20%; Δ*L*_f_: free linear displacement, *L*_0_: initial length) that necessitates operation at high, near‐resonant frequencies (up to 1000 Hz) for navigation.^[^ ^1^, ^3^, ^5^, ^7^ ^]^ While microrobots powered by soft mDEAs demonstrate swift movements in manmade environments characterized by smooth surfaces, their mobility is still compromised in environments with obstacles exceeding the actuator's displacement. Their mobility is further limited in fluidic environments, where viscous forces dominate and inertial forces diminish at the microscale, making propulsion particularly challenging for actuators with small displacement operating at high frequencies. Since the ability to navigate diverse environments, especially unstructured ones, is crucial for microrobots, it is necessary to develop mDEAs with larger linear displacements by incorporating soft–stiff hybrid designs.

In parallel with actuator development, realizing the full potential of modular design also requires a broad range of modules that are individually optimized for specific tasks. However, the fabrication of mesoscale components compatible with microrobots often relies on custom‐designed processes and specialized equipment,^[^ ^1^, ^2^, ^3^ ^]^ posing challenges such as increased design and fabrication costs, reduced system flexibility, and module compatibility issues. A broadly applicable fabrication strategy that enables the production of mesoscale components is therefore expected to create synergy with microrobots featuring a modular design.

**Figure 1:**
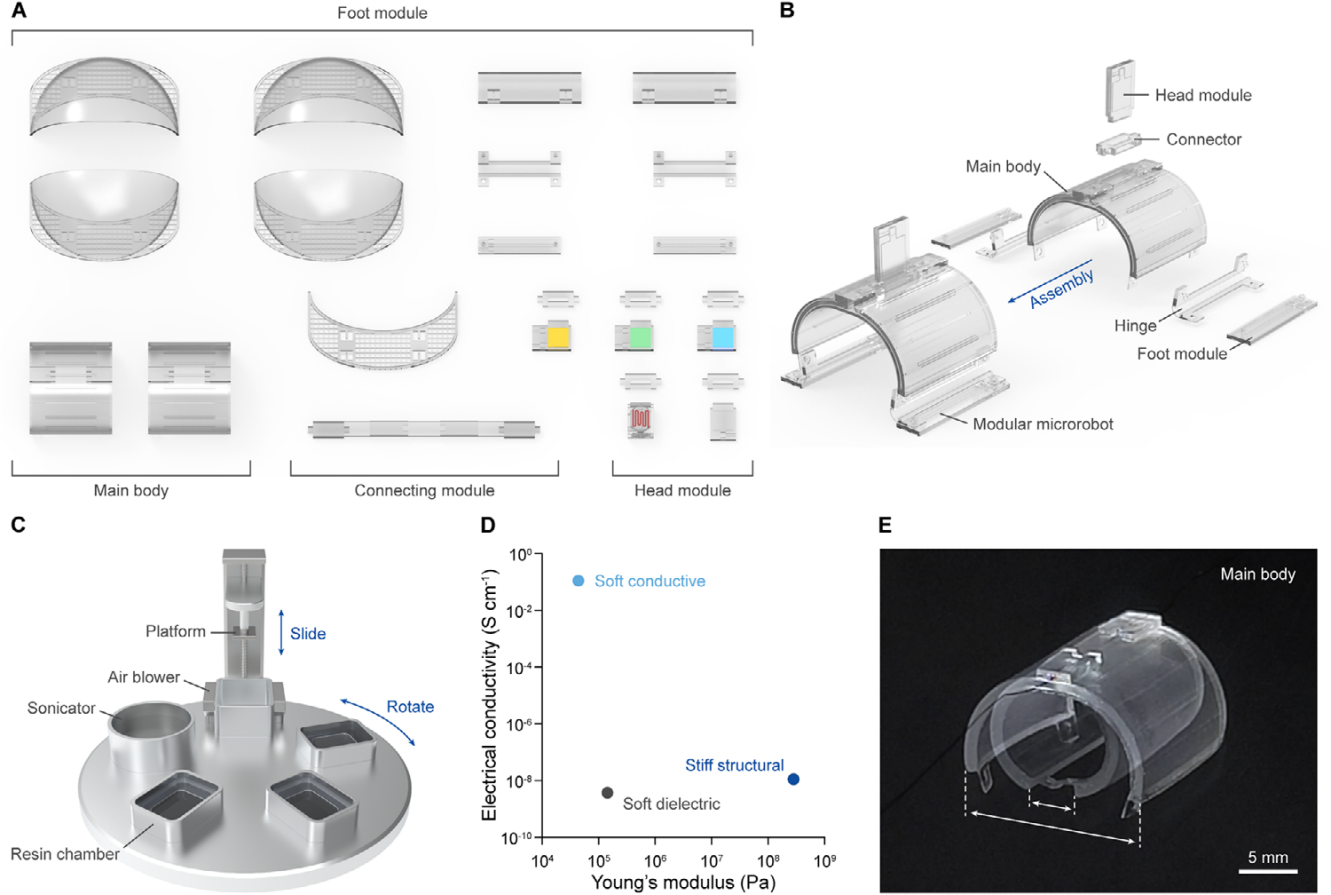
Modular design and mesoscale multimaterial fabrication strategies for microrobots. A) Schematics of the main bodies and ten modules demonstrated in this study. A modular design facilitates efficient structural and functional adaptation by subdividing the microrobot into function‐specific components: the main body, foot modules, head modules, and connecting modules. B) Assembly of the modular microrobot. The basic unit of the modular microrobot consists of the main body equipped with two foot modules and one head module. The interlocking design enables reversible assembly between the main body and the modules. C) Schematic of the digital light processing multimaterial (DLPM) 3D printer developed to fabricate a broad range of mesoscale components essential for the modular design. The printer can simultaneously print three distinct photocurable resins and features an automated cleaning system with a sonicator and air blower to prevent cross‐contamination between resins. D) Young's modulus and electrical conductivity of the three photocurable resins used to fabricate the main body and the modules demonstrated in this study. E) Photograph of the main body powered by two large‐displacement dielectric elastomer microactuators (mDEAs). The main body generates substantial mechanical movement to overcome obstacles on land and propels the robot rapidly on water. An electric field strength of 16 kV mm^−1^ was applied during operation. Scale bar, 5 mm.

Here, we report fully 3D‐printed modular microrobots capable of navigating multiple environments, interacting with other robots and humans in real time, and performing demanding tasks through multi‐unit collaboration. (**Figure** **1**A,B). To fabricate the broad range of mesoscale components required for the microrobots, we developed a digital light processing multimaterial (DLPM) 3D printer^[^ ^8^ ^]^ specifically optimized for microrobotic applications. The printer can simultaneously print various photocurable resins, including conductors, insulators, structural materials, and functional materials, with a resolution of 10 µm (Figure 1C,D; Figures S1 and S2, and Video S1, Supporting Information). Building on this mesoscale multimaterial printing capability, we designed large‐displacement mDEAs incorporating the soft–stiff hybrid structure to enable navigation in various unstructured environments. In parallel, we developed a high‐performance soft dielectric resin with tailored electromechanical properties and self‐swelling behavior to further enhance its linear displacement. The resulting 3D‐printed mDEAs exhibited significantly enhanced linear displacement (|Δ*L*_f_|/*L*_0_ = 145.3%) even at low, non‐resonant frequencies, providing sufficient mechanical output to stride over obstacles on various terrestrial terrain and rapidly propel themselves across aquatic terrain (Figure 1E). Consequently, the microrobots achieve swift navigation across smooth (*v* = 21.8 BL min^−1^; *v*: velocity, BL min^−1^: body lengths per minute), rough (*v* = 17.1 BL min^−1^), granular (*v* = 13.4 BL min^−1^), and aquatic (*v* = 53.2 BL min^−1^) environments. To further expand the capabilities of the microrobots, we designed and fabricated ten types of modules, including sensors, structural elements, and communication units, each optimized for a specific task. The 3D‐printed modules enabled the microrobots to avoid collisions through noncontact sensing of nearby humans and to control the movements of other microrobots via optical signaling. In addition, by utilizing the synergy between modular design and DLPM 3D printing technology, which enables the rapid and efficient mass production of the modules, we demonstrated multi‐unit collaboration. Effective collaboration among multiple units allows the robots to surpass the performance limits of a single‐unit robot by significantly increasing power (dragging objects 100 times their body weight), enhancing speed (up to 50%), and enabling swift directional control (ω = 28.7° s^−1^; ω: angular velocity).

## 2 Results and Discussion

### 2.1 Large‐Displacement mDEAs for Main Body

**Figure 2:**
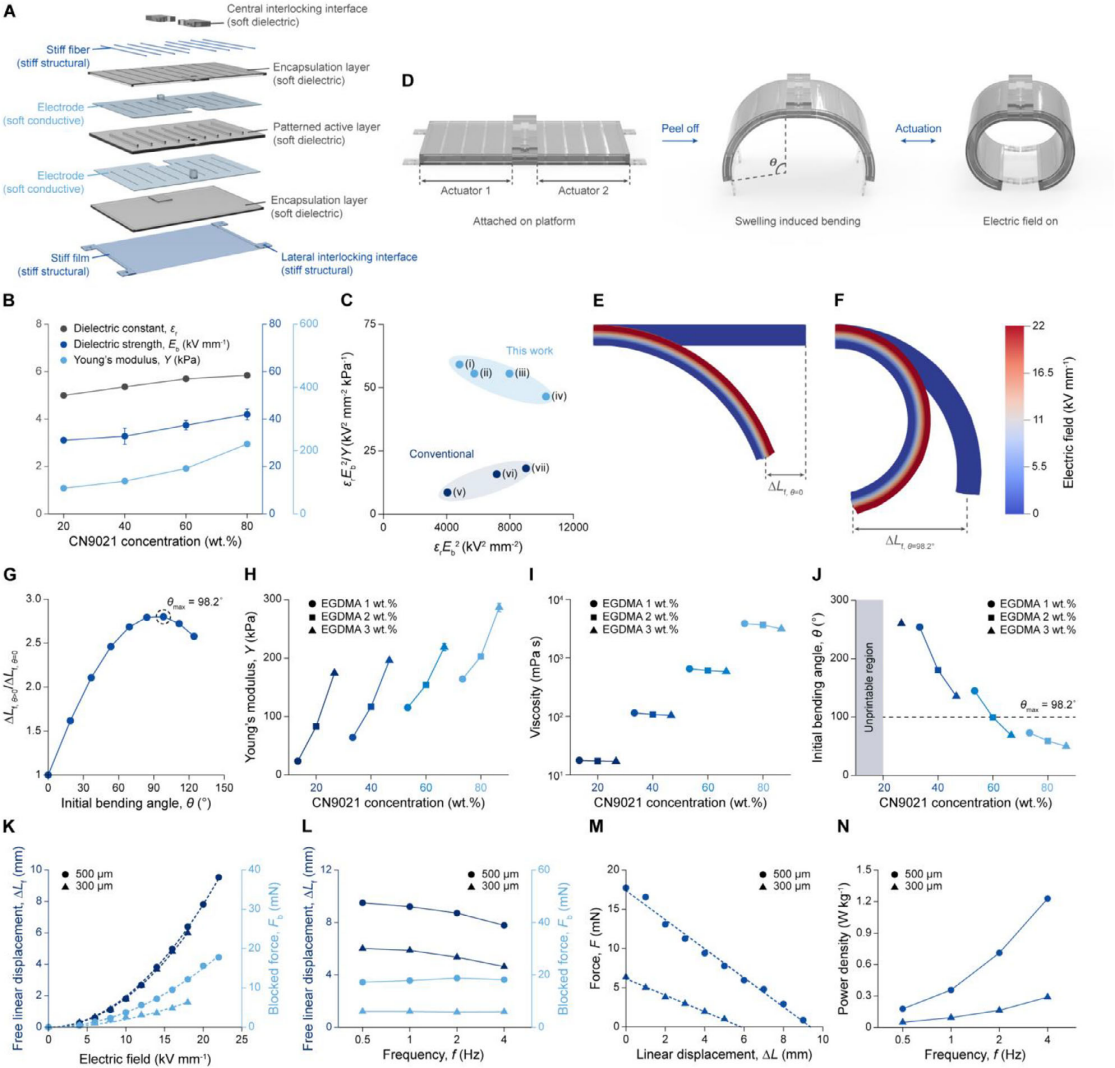
Large‐displacement mDEAs for main body. A) Exploded view of the 3D‐printed main body powered by two large‐displacement mDEAs featuring a soft‐stiff hybrid design. The stiff film and fibers, combined with 3D patterns on active layer, efficiently convert the small in‐plane areal expansion of active layer into large out‐of‐plane bending of the actuators. B) Electromechanical properties of the soft dielectric resin as a function of CN9021 concentration, including the dielectric constant (ε_r_), dielectric strength (*E*_b_), and Young's modulus (*Y*). Error bars indicate SDs; N = 3. C) Ashby plot comparing the maximum stress (proportional to ε_r_ *E*_b_^2^) and maximum strain (proportional to ε_r_ *E*_b_^2^/*Y*) of the developed soft dielectric resins with those of conventional soft dielectric resins for digital light processing (DLP) 3D printing applications. The developed resin with CN9021 concentrations of i) 20 wt.%, ii) 40 wt.%, iii) 60 wt.%, and iv) 80 wt.% was compared with conventional resins, including v) CYKE05C, vi) FLX930, and vii) KER‐4303‐UV. D) Schematics of configuration changes of the main body. Initially printed in a flat form, the mDEA bends to an optimal angle upon peeling from the printing platform due to the internal stress formed by the self‐swelling of the soft dielectric resin. The initial bending angle (θ) is defined as the bending angle of the actuator in its resting state. The application of electric field causes further bending by actuation. E–G) Finite element method (FEM) simulations showing the effect of the initial bending angle on the free linear displacement (Δ*L*_f_) of mDEAs. Simulation results of the mDEA before and after applying an electric field, with an initial bending angle of (E) 0° and (F) 98.2°. The electric field strength applied to the model is represented by color. (G) Simulated free linear displacement of the self‐swelled, bent (θ > 0°) mDEA relative to the unswelled, flat (θ = 0°) mDEA (Δ*L*_f, θ > 0_/Δ*L*_f, θ  =  0_) as a function of the initial bending angle. H,I) Effect of soft dielectric resin composition on the (H) Young's modulus of the cured polymer and (I) viscosity of the precursor solution. Error bars indicate SDs; N = 3. J) Initial bending angle of the mDEA induced by self‐swelling during 3D printing, as a function of soft dielectric resin composition. Dashed lines indicate the optimal initial bending angle (θ_max_) obtained through simulations. K–N) Actuation performance of mDEAs measured for active layer thicknesses of 500 µm and 300 µm. K) Blocked force (*F*_b_) and free linear displacement as functions of the electric field strength applied to the active layer. L) Blocked force and free linear displacement in the low, non‐resonant frequency range (*f*), expected to be used in the microrobot. M) Relationship between force (*F*) and linear displacement (Δ*L*). N) Power density as a function of operating frequency.

The microrobot, which employs modularity for efficient structural and functional adaptation, consists of a main body and three types of modules—foot, head, and connecting modules—based on their functions (Figure 1A). Among these, the main body serves as a central platform, hosting other modules and controlling the mechanical movement of the microrobot. With an interlocking design, the modules can be reversibly assembled to the main body (Figure 1B). For multi‐environment navigation, the main body incorporated two large‐displacement mDEAs featuring a soft‐stiff hybrid design (**Figure** **2**A; Figure S3 and Video S2, Supporting Information). At the center, an active layer was sandwiched between two electrodes. To prevent electrical breakdown between the electrodes and modules assembled to the main body, cover layers were introduced for encapsulation. A thin stiff film was placed at the bottom of the actuator to convert the in‐plane areal expansion of the active layer into out‐of‐plane bending of the actuator.^[^ ^6a,d^ ^]^ Two lateral interlocking interfaces for the foot modules were placed on either side of the stiff film. Since the stiff film alone cannot precisely control the bending direction, stiff fibers were added at the top of the actuator to restrict bending in unwanted directions (Figure S4 and Video S3, Supporting Information).^[^ ^6^, ^9^ ^]^ To facilitate efficient force transfer between the active layer and the stiff elements, 3D patterns in the form of horizontally aligned walls were added to both sides of the active layer. Finally, a central interlocking interface for the head modules was added above the stiff fibers.

To realize the soft‐stiff hybrid design of the mDEAs, the DLPM 3D printer, capable of simultaneously printing all required materials—soft dielectric, soft conductive, and stiff structural resins—was employed to fabricate the main body (Figure 1D; Figure S1, Supporting Information). Among the materials comprising the mDEA, the soft dielectric resin especially critical role in determining the actuation performance. The maximum stress (σ_max_) and maximum strain (*S*_max_) that a mDEA can achieve before electrical breakdown is determined by the dielectric constant (ε_r_), dielectric strength (*E*_b_), and Young's modulus (*Y*) of the soft dielectric material composing the active layer:^[^ ^5^, ^10^ ^]^

(1) $$\sigma_{\max}\approx \varepsilon_{r}{E_{b}}^{2}$$

(2) $$S_{\max}\approx \frac{\varepsilon_{r}{E_{b}}^{2}}{Y}$$

Based on this relationship, we developed a soft dielectric resin optimized for large displacement by tailoring its electromechanical properties. First, we selected 2‐ethylhexyl acrylate (EHA) as the monomer for the soft dielectric resin due to its low viscosity and vapor pressure. The low viscosity (*η* = 1.67 mPa s at 20 °C) facilitated the cleaning of residual resin on printed surfaces, minimizing cross‐contamination between resins during multimaterial printing. The low vapor pressure (*P*_vap_ = 20 Pa at 20 °C) minimized the strong odor of acrylate monomers, making the resin more suitable for 3D printing applications. To enhance the electrical properties of the resin, CN9021, a high molecular weight ($\bar{M_{n}}$ = 28 000 g mol^−1^) urethane diacrylate compound with a flexible polyether diol segment and an aliphatic diisocyanate segment, was used as a long‐chain cross‐linker.^[^ ^5^, ^10^ ^]^ As the concentration of CN9021 increased, the dielectric constant and dielectric strength of the soft dielectric resin both increased (Figure 2B). Ethylene glycol dimethacrylate (EGDMA) was added as a short‐chain cross‐linker to increase the curing speed of the resin for high‐resolution printing, while diphenyl(2,4,6‐trimethylbenzoyl)phosphine oxide (TPO) was used as a photoinitiator. The maximum stress (proportional to ε_r_ *E*_b_^2^) and maximum strain (proportional to ε_r_ *E*_b_^2^/*Y*) of the developed resin with varying monomer and long‐chain cross‐linker concentrations were compared to those of conventional soft dielectric resins for digital light processing (DLP) 3D printing applications (Figure 2C; Figure S5, Supporting Information). Our soft dielectric resin maintained a high maximum stress while exhibiting a substantially higher maximum strain than conventional resin across all compositions, suggesting its potential to greatly enhance linear displacement in mDEAs.

The initial bending angle (θ), defined as the bending angle of the actuator in its resting state (Figure 2D), is an important factor that significantly affects the linear displacement of bending‐type DEAs.^[^ ^6a,d^ ^]^ Previously, to achieve the initial bending, the active layer is often prestretched before being combined with the stiff elements.^[^ ^6a,d^ ^]^ However, this prestretching method is challenging to integrate with 3D printing. To achieve the optimal initial bending of the mDEA fabricated using the DLPM 3D printer, we carefully controlled the self‐swelling of the soft dielectric resin. In photopolymerization‐based 3D printing methods like DLP or stereolithography (SLA), the cured polymer remains submerged in the precursor solution to enable printing of the next layer. During this process, the cured polymer swells as it absorbs its precursor solution. This phenomenon, known as self‐swelling, typically occurs in soft resins with a low Young's modulus in the kilopascal range and is often considered undesirable because it can distort the printed structure (Figure S6, Supporting Information). So far, efforts have focused on mitigating self‐swelling by increasing the viscosity of the precursor solution or the Young's modulus of the cured polymer. However, we anticipated that controlled self‐swelling could induce initial bending in 3D‐printed mDEAs without the need for active layer prestretching. Among the resins used in the mDEA, we focused on the soft dielectric resin because its self‐swelling is more pronounced than that of the stiff structural resin—with a Young's modulus 2000 times higher—or the soft conductive resin—which contains over 85 wt.% solvent even before self‐swelling. We first investigated whether sufficient initial bending could be induced solely by self‐swelling of the soft dielectric resin during the printing process through finite element method (FEM) simulations. The simulation results revealed that large initial bending angles exceeding 120° could be achieved within a few minutes—the time typically required to print each layer (Figures S6 and S7, Supporting Information). Furthermore, when an electric field is applied, the self‐swelled, bent (θ > 0°) mDEA was predicted to exhibit up to a 180% increase in free linear displacement compared to the unswelled, flat (θ = 0°) mDEA (Δ*L*_f, θ > 0_/Δ*L*_f, θ  =  0_ = 2.8 at θ = 98.2^°^) (Figure 2E–G).

To achieve an initial bending angle close to the simulated optimum, we fine‐tuned the viscosity and Young's modulus of the soft dielectric resin. The Young's modulus of the cured polymer was adjusted using a short‐chain cross‐linker concentration (Figure 2H), while the viscosity of the precursor solution was modified through a long‐chain cross‐linker concentration (Figure 2I). Through varying cross‐linker compositions, the initial bending angle of the 3D‐printed mDEA was adjusted across a wide range, from 49.7° to 260.5° (Figure 2J). The composition of CN9021 60 wt.% and EGDMA 2 wt.% was selected for mDEA fabrication, as it induced a 99.4° initial bending angle that was the closest to the optimal angle (θ_max_) of 98.2° predicted by the FEM simulations to maximize linear displacement (Figure 2G; Figure S8, Supporting Information). As a result, the mDEA, initially printed in a flat configuration, bent to an optimal angle after being peeled from the printing platform due to the internal stress formed by the self‐swelling of the soft dielectric resin. When an electric field is applied, the in‐plane expansion of the active layer is converted into out‐of‐plane bending through the stiff elements, resulting in further bending (Figure 2D). During actuation, the 3D patterns in the active layer enable efficient force transfer between the active layer and the stiff elements, leading to an increase in free linear displacement of more than 38.5% (Figure S9, Supporting Information).

To evaluate the actuation performance of the printed bending‐type mDEA, the blocked force (*F*_b_) and free linear displacement were measured as functions of the electric field strength applied to the active layer (Figure 2K; Figure S10, Supporting Information). As the electric field strength increased, both the blocked force and free linear displacement increased in proportion to the square of the field strength. With an active layer thickness of 500 µm, the 6.52 mm long actuator generated a maximum blocked force of 17.78 mN and a free linear displacement of 9.53 mm, before undergoing electrical breakdown at an electric field strength of 22 kV mm^−1^. However, when the active layer thickness was reduced to 300 µm, the mDEA experienced electrical breakdown at 18 kV mm^−1^ (Figure S11, Supporting Information). This was due to cross‐contamination between resins during the multimaterial printing process, causing the effective thickness of the active layer to be less than the design (see Supporting Information Text). To analyze the actuation performance in the low, non‐resonant range expected to be used by the microrobot, the blocked force and free linear displacement of the actuator were measured across the operating frequency (*f*) range of 0.5 to 4 Hz (Figure 2L). The actuator exhibited stable performance across the operating frequency range, maintaining consistent blocked force while the free linear displacement decreased by only about 18%, making it ideal for powering the main body of the modular microrobot. To further evaluate the actuation performance of the printed mDEA, the relationship between force (*F*) and linear displacement (Δ*L*) was measured. The force of the actuator decreased linearly with increasing linear displacement (Figure 2M). Based on the measured relationship between force and linear displacement, the energy density (*e*) and power density (p) output of the actuator can be calculated as:^[^ ^3c^ ^]^

(3) $$e=\frac{F_{b}\Delta L_{f}}{2m}$$

(4) $$p=\frac{F_{b}\Delta L_{f}f}{2m}$$

where, *m* is the mass of the actuator. The energy density that the mDEA could perform decreased by 14% as the frequency increased from 0.5 to 4 Hz, while the power density rose by 591% (Figure 2N; Figure S12, Supporting Information). The long‐term stability of the printed mDEA was verified through cyclic testing, where it demonstrated stable performance over 5000 cycles at a frequency of 4 Hz for a total of 1250 s (Figure S13, Supporting Information). In conclusion, the linear displacement of mDEAs with a soft‐stiff hybrid design exceeded that of conventional mDEAs made entirely of soft materials, even at low operating frequencies outside of resonance, thereby broadening their potential applications across diverse environments.^[^ ^1^, ^3^, ^5^, ^7^ ^]^

### 2.2 Foot Modules for Multi‐Environment Navigation

**Figure 3:**
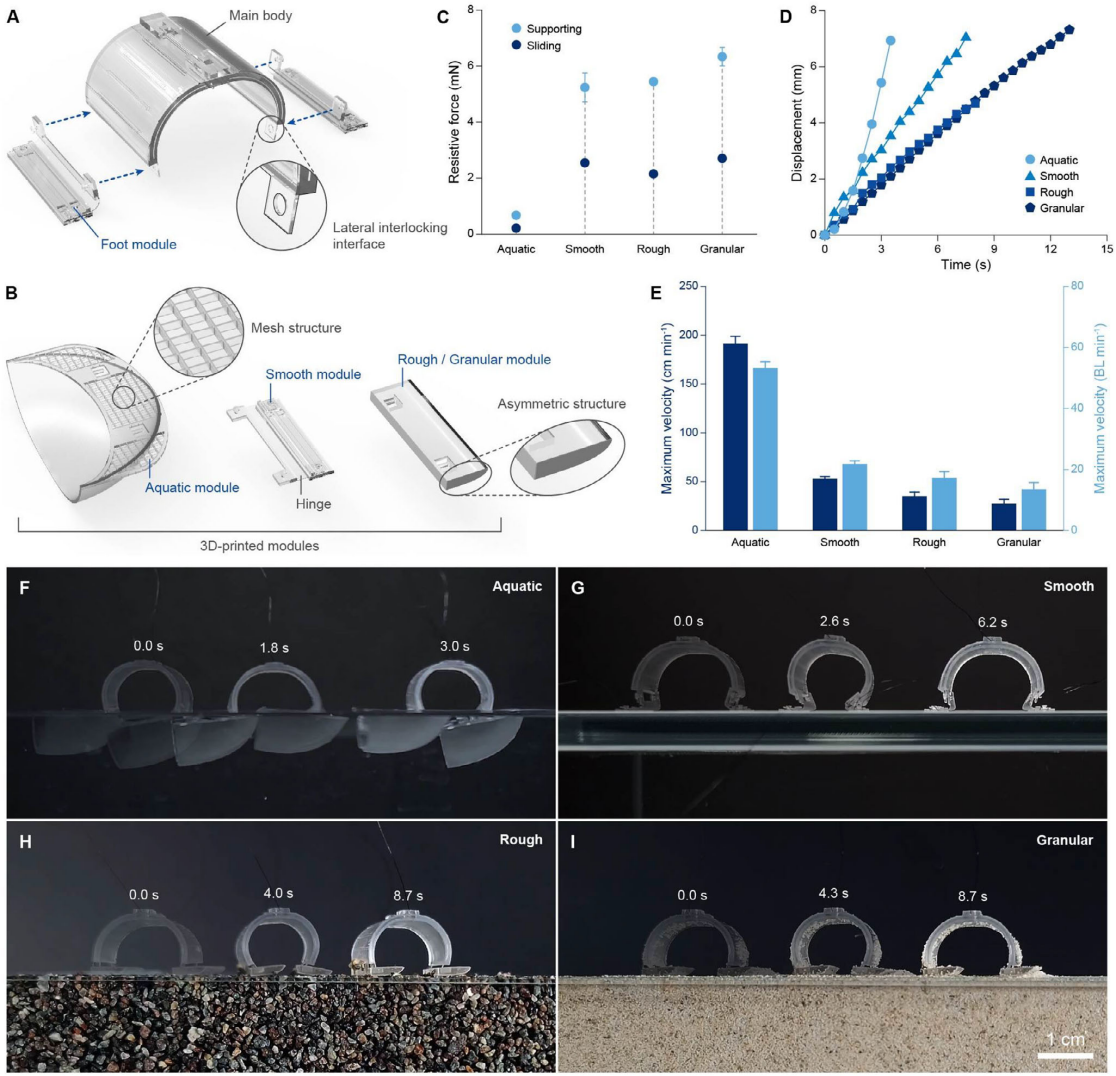
Foot modules for multi‐environment navigation. A) Assembly of the foot modules onto the main body. Foot modules are reversibly assembled onto the two lateral interlocking interfaces located at either end of the main body. B) Schematics of the three foot modules designed for navigating four environments: aquatic, smooth, rough, and granular. Each foot module converts the symmetrical actuation of the main body into directional movement of the robot, tailored to its respective environment. C) Resistive force of the foot modules during the supporting and sliding phase. When the main body contracts, the front foot enters the supporting phase while the rear foot slides forward. Conversely, when the main body extends, the rear foot enters the supporting phase, and the front foot slides forward. Error bars indicate SDs; N = 3. D) Displacement of the modular microrobot over time in various environments. The microrobot accelerates gradually in aquatic environments while maintaining a constant velocity in terrestrial environments. E) Maximum velocity of the microrobot in various environments. Variations in body length, dependent on the assembled foot modules, result in differences between absolute velocity and body‐length‐normalized velocity. Error bars indicate SDs; N = 3. F–I) Time‐lapse images of the microrobot navigating various environments: F) aquatic, G) smooth, H) rough, and I) granular environments. Scale bar, 1 cm.

The foot modules, reversibly assembled at the lateral interlocking interfaces located at each end of the main body (**Figure** **3**A; Figure S14, Supporting Information), were designed to transform the symmetrical actuation of the main body into directional movement of the robot, by generating anisotropic resistive forces (e.g., friction and drag) across smooth, rough, granular, and aquatic environments (Figure S15, Supporting Information). As the main body contracts, the resistive force counteracting the backward pull on the front foot is stronger than that counteracting the forward pull on the rear foot, making the front foot provide support while the rear foot slides forward. Conversely, as the main body extends, the resistive force counteracting the forward push on the front foot is weaker than that counteracting the backward push on the rear foot, making the rear foot provide support while the front foot slides forward. Accordingly, the three types of foot modules employ distinct strategies, enabling the microrobot to adapt seamlessly across four different environments (Figure 3B). Designed for aquatic locomotion, the aquatic module has a top mesh that keeps the robot afloat by surface tension of water,^[^ ^2^, ^11^ ^]^ while submerged webbing below creates directional drag to propel the microrobot forward. On smooth surfaces, the smooth module utilizes electrostatic adhesion to control friction with the surface, enabling forward and backward movement through adjustments in the voltage sequence applied to the main body and foot module (Figures S16 and S17, and Supporting Information Text, Supporting Information).^[^ ^1^, ^6^, ^12^ ^]^ To ensure consistent ground contact, a hinge mechanism was implemented to keep the foot module parallel to the surface, regardless of the main body's actuation. The rough/granular module with an asymmetric structure—angled in the back and curved in the front—achieves directional friction, allowing the microrobot to adapt to both rough environments composed of coarse sand (particle size ≈1.5 mm) and granular environments made up of fine sand (particle size ≈0.15 mm). To evaluate the anisotropic friction or drag characteristics of each foot module, resistive force was measured during both supporting and sliding phases. Across all modules, the resistive forces varied by more than twofold depending on the phase, providing sufficient directional friction or drag to enable the microrobot to navigate across multiple environments (Figure 3C; Figure S18, Supporting Information).

The microrobot's adaptability across various environments was demonstrated by equipping each foot module (Figure 3D; Video S4, Supporting Information). In the aquatic environment, the microrobot accelerated gradually and reached a maximum velocity of 53.2 BL min^−1^. Among terrestrial environments, it moved fastest on smooth surfaces at 21.8 BL min^−1^, while on rough and granular surfaces, it achieved 17.1 and 13.4 BL min^−1^, respectively (Figure 3E; Table S1, Supporting Information). To further understand the microrobot's locomotion, its interactions with each environment were observed (Video S4, Supporting Information). In the aquatic environment, the foot modules exhibited repetitive back‐and‐forth rotation due to strong drag forces counteracting the main body's actuation. Despite these rotations, the microrobot remained afloat, supported by the high surface tension provided by the mesh structure of the aquatic module (Figure 3F). On smooth surfaces, the microrobot smoothly reversed its walking direction by adjusting the voltage sequence applied to the foot modules and main body (Figure 3G; Figure S17, Supporting Information). In the rough environment, the coarse sand exhibited negligible movement (Figure 3H), whereas in the granular environment, fine sand accumulated in front of the microrobot as it walked forward (Figure 3I).

### 2.3 Head Modules for Real‐Time Interaction

**Figure 4:**
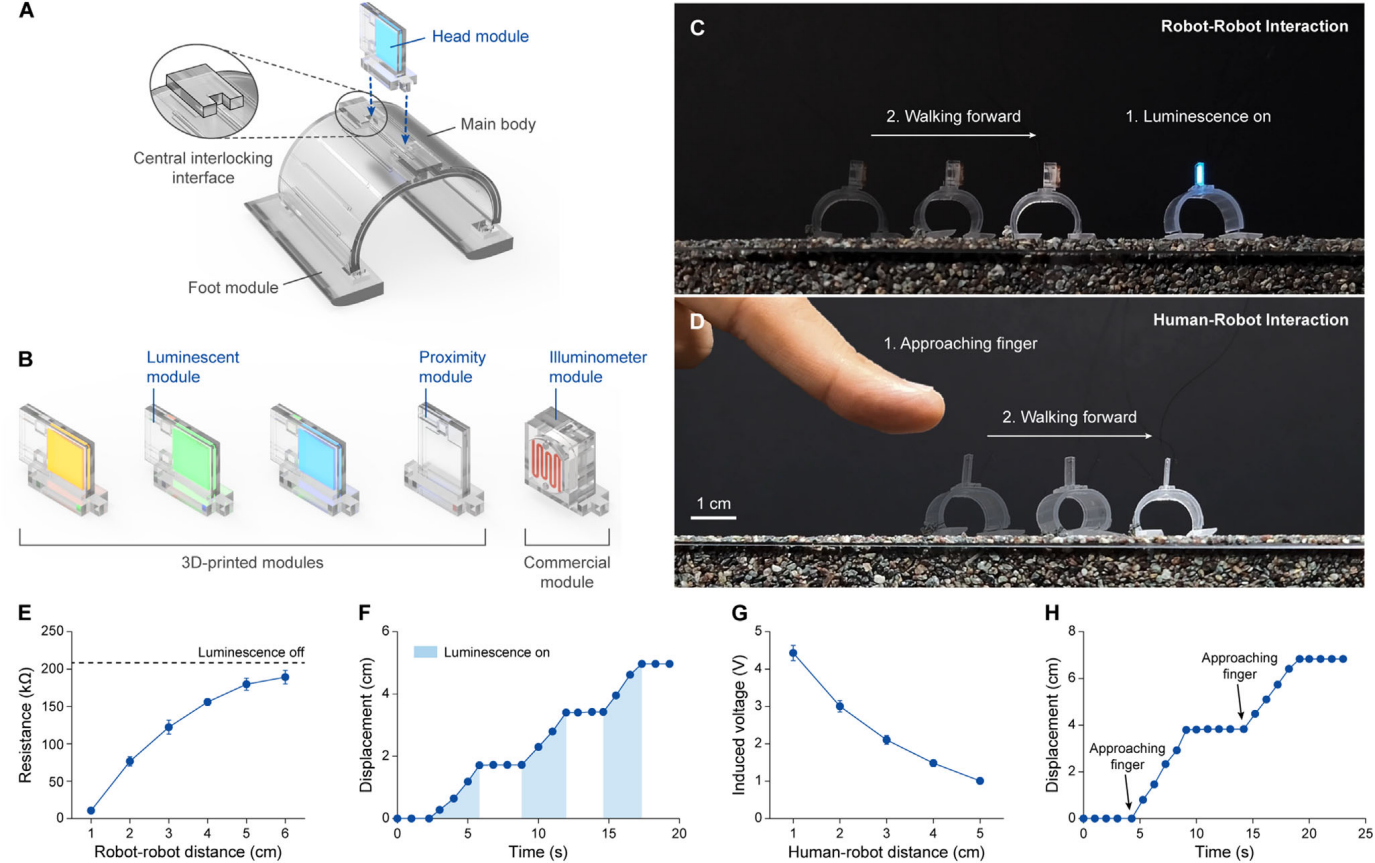
Head modules for real‐time interaction. A) Assembly of the head modules to the main body. The head modules are reversibly assembled onto the central interlocking interface located at the center of the main body. B) Schematics of the three types of head modules developed for real‐time interaction with other robots and humans. Luminescent modules of varying colors and the proximity module were fabricated using DLPM 3D printing. The illuminometer module, developed by integrating a commercial photoresistor with a 3D‐printed connector, demonstrates compatibility with components fabricated using methods other than 3D printing. C,D) Time‐lapse images of microrobots demonstrating real‐time robot‐robot interaction (C) and human–robot interaction (D). Scale bar, 1 cm. E) Resistance change of the illuminometer module as a function of the distance between the leader and follower robots. The illuminometer module allows the follower robot to detect optical signals emitted by the luminescent module on the leader robot. Error bars indicate SDs; N = 3. F) Displacement of the follower robot over time during the demonstration. The follower robot walked forward only when the luminescent module on the leader robot was activated. G) Induced voltage in the proximity module as a function of the distance between the robot and a human finger. The proximity module allows the robot to detect the approach of a human finger without physical contact. Error bars indicate SDs; N = 3. H) Displacement of the robot over time during the demonstration. The robot walked forward a set distance to avoid collisions whenever a finger approached.

The head module, reversibly assembled at the central interlocking interface located at the center of the main body, extends the microrobot's capabilities beyond mere navigation, enabling real‐time interaction with its environments (**Figure** **4**A). Three types of head modules were developed to demonstrate interaction with other robots and humans (Figure 4B). The luminescent module, designed for optical signaling, controls its brightness and color through the applied electric field strength and the type of electroluminescent phosphor used in the luminescent layer, respectively (Figure S19, Supporting Information).^[^ ^13^ ^]^ The proximity module senses the approach of nearby objects by measuring the induced voltage in the receiver, providing an estimate of relative distances (Figure S20, Supporting Information).^[^ ^12^, ^14^ ^]^ Additionally, to demonstrate compatibility with components fabricated through methods other than DLPM 3D printing, the illuminometer module—designed to measure ambient light intensity—was developed by integrating a commercial photoresistor with a 3D‐printed connector that assembles to the central interlocking interface for head modules (Figure S21, Supporting Information).

The microrobot's real‐time interactions with other robots and humans were demonstrated in rough environments (Figure 4C,D). For robot–robot interaction, a leader robot equipped with a luminescent module used optical signals to direct a follower robot equipped with an illuminometer module on when to move. In standby mode, the illuminometer module on the follower robot exhibited a resistance of 208.6 kΩ. Upon activation of the luminescent module on the leader robot, the illuminometer module detected optical signals from several centimeters away, with its resistance decreasing by over 9.3% (Figure 4E). Using this communication mechanism, the follower robot was programmed to walk forward only when the luminescent module was activated, enabling the leader robot to control the follower's movement in real time (Figure 4F; Video S5, Supporting Information). For human–robot interaction, a microrobot equipped with a proximity module successfully detected human presence and avoided collisions. In the absence of nearby humans, the induced voltage in the proximity module remained at zero. However, as a human finger approached, a voltage exceeding 1.00 V was induced, demonstrating its ability to detect humans several centimeters away without physical contact (Figure 4G). Leveraging this detection capability, the microrobot was programmed to automatically walk forward a set distance to avoid collisions whenever a finger approached, successfully executing multiple instances of real‐time collision avoidance (Figure 4H; Figure S22 and Video S6, Supporting Information).

### 2.4 Connecting Modules for Multi‐Unit Collaboration

**Figure 5:**
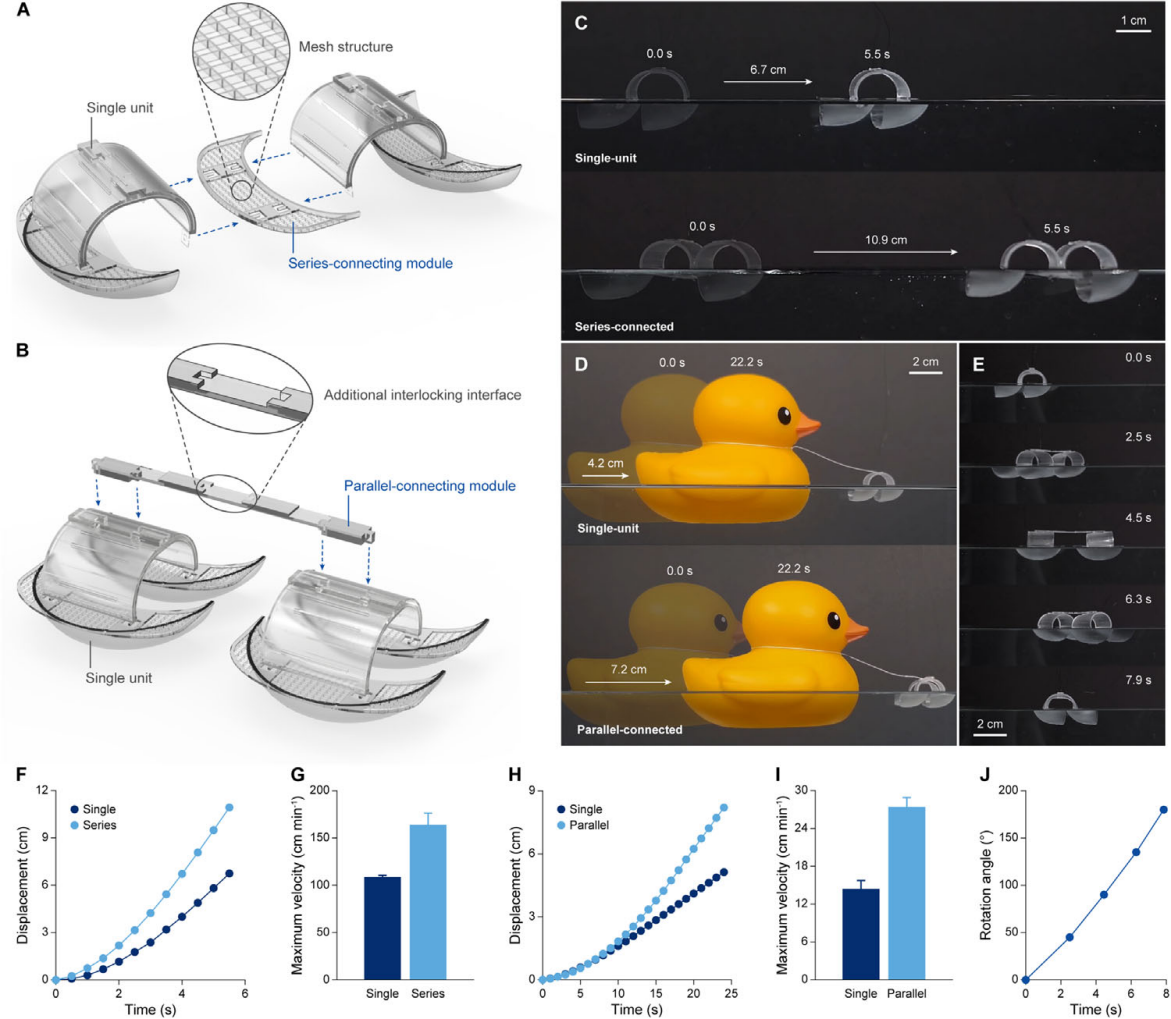
Connecting modules for multi‐unit collaboration. A) Series connection of two units using a series‐connecting module. The module assembles to one of the lateral interlocking interfaces on each unit's main body, replacing a foot module. B) Parallel connection of two units using a parallel‐connecting module. The module assembles to the central interlocking interfaces on each unit's main body, replacing a head module. It includes an additional interlocking interface to enable the head module to be assembled to the parallel‐connected robot. C–E) Time‐lapse images of microrobots demonstrating increased speed through series connection (C), enhanced force through parallel connection (D), and directional adjustment through parallel connection (E). In series connection demonstration, a sinusoidal electric field with an amplitude of 6 kV mm^−1^ and an offset of 6 kV mm^−1^ was applied to each unit. Scale bars, 1 cm (C); 2 cm (D, E). F) Displacement of a single‐unit robot and a series‐connected robot over time. G) Maximum velocities of the single‐unit robot and the series‐connected robot. Error bars indicate SDs; N = 3. H) Displacement of a single‐unit robot and a parallel‐connected robot pulling a rubber duck over time. The rubber duck weighs 110 g, equivalent to 100 times the weight of the single‐unit robot. I) Maximum velocities of the single‐unit robot and the parallel‐connected robot pulling a rubber duck. Error bars indicate SDs; N = 3. J) Rotation angle of the parallel‐connected robot over time.

Introducing DLPM 3D printing technology into the fabrication of modular microrobots not only enabled the production of diverse mesoscale components but also allowed for their rapid and efficient mass production (Video S1, Supporting Information). Therefore, by harnessing the synergy of DLPM 3D printing technology and modular design, demanding tasks beyond the capabilities of single‐unit robots were achieved through the collaboration of multiple units. To facilitate seamless collaboration, a connecting module was developed, offering two distinct configurations: one optimized for enhanced speed and the other for enhanced force output and control. The series‐connecting module, assembled at the lateral interlocking interfaces, links units in series to increase step length, thereby enhancing navigation speed (**Figure** **5**A). In contrast, the parallel‐connecting module, assembled at the central interlocking interface, links units in parallel; synchronized control of the units enhanced force output, while independent control of each unit allowed for directional adjustments (Figure 5B). An additional interlocking interface was added to the center of the parallel‐connecting module to enable the assembly of a head module to the parallel‐connected robot.

The performance of the multi‐unit robot in both series and parallel configurations was evaluated in aquatic environments (Figure 5C–E; Videos S7 and S8, Supporting Information). The series‐connected robot demonstrated superior speed, traveling 10.9 cm in 5.5 s, while the single‐unit robot covered only 6.7 cm in the same duration (Figure 5C). Its extended step length allowed the series‐connected robot to accelerate faster, achieving a maximum velocity 50% greater than that of the single‐unit robot (Figure 5F,G). Similarly, the parallel‐connected robot demonstrated an increased force output by pulling a 110 g rubber duck, which is equivalent to 100 times the weight of the 1.1 g single‐unit robot. The parallel‐connected robot covered 7.2 cm in 22.2 s, a 71.4% increase in distance compared to the single‐unit robot over the same duration (Figure 5D). During this demonstration, the parallel‐connected robot gradually pulled ahead, reaching a maximum velocity 90% greater than that of the single‐unit robot (Figure 5H,I). In addition to its force output, the parallel‐connected configuration allowed for swift directional control (Figure 5E). Activating only one of the two parallel‐connected units enabled the robot to perform a 180° turn in just 7.9 s, with the stationary unit serving as a pivot (Figure 5J). During the rotation, the robot's angular velocity increased progressively, reaching a peak of 28.7° s^−1^.

## 3 Conclusion

In this study, we demonstrated the potential of modular microrobots by addressing two major challenges that have hindered their development: the absence of large‐displacement microactuators suitable for navigation across diverse environments, and the need for a wide range of mesoscale modules to perform practical tasks beyond mere navigation. To overcome the first challenge, we developed dielectric elastomer microactuators that exhibited significantly enhanced linear displacement by incorporating a soft–stiff hybrid design and a high‐performance soft dielectric resin with tailored electromechanical properties and self‐swelling behavior. To address the second, we developed a digital light processing multimaterial 3D printer optimized for microrobotic applications, enabling the fabrication of all ten types of modules developed in this study. With these components, the microrobots successfully operated across a wide range of environments, including smooth, rough, granular, and aquatic terrains. Moreover, they performed tasks such as real‐time control of neighboring robots through optical signaling, autonomous collision avoidance with approaching humans via proximity sensing, and transport of heavy objects up to 100 times the weight of a single‐unit robot through multi‐unit collaboration. While demonstrating potential for practical applications, current modular microrobots still require manual replacement of modules to adapt to new tasks and environments, and rely on external power sources and computing circuits connected by wires. Therefore, future studies on automated module replacement and the integration of onboard batteries and computing circuits for untethered operation are expected to further improve their adaptability to complex and diverse situations encountered in practical applications.

## 4 Experimental Section

### DLPM 3D Printer Setup

A DLPM 3D printer capable of simultaneously printing up to three photocurable resins was custom‐built to fabricate the mesoscale components required for modular microrobots (Figure 1D; Video S1, Supporting Information). The printer consists of three main sections. At the bottom, an optical system directs UV light onto selected regions. In the middle, a rotating disk houses three resin chambers, a sonicator, and an air blower. At the top, the printing platform is located. The optical system at the bottom and the printing platform at the top are vertically aligned, while the rotating disk in the middle positions the resin chambers, the sonicator, or the air blower between the optical system and the printing platform as needed.

The optical system comprises a 365 nm UV light‐emitting diode (LED) and a digital micromirror device (DMD) with a resolution of 2716 pixels × 1528 pixels. The UV intensity was adjusted between 0.1 and 0.6 W cm^−^^2^ depending on the resin. The UV light emitted by the LED is selectively reflected by the DMD and directed to the resin chamber. On the rotating disk, the three resin chambers, sonicator, and air blower are arranged at 72° intervals. The bottom of each resin chamber is made of fluorinated ethylene propylene film, designed to effectively transmit UV light to the resin while allowing the cured material to detach easily and adhere to the printing platform. The sonicator, containing isopropyl alcohol (IPA), performs the first cleaning step by removing uncured resin from the printing platform. The air blower uses high‐pressure nitrogen gas to remove residual IPA and any remaining uncured resin during the second cleaning step. The printing platform was designed to slide vertically, enabling height adjustments in 10 µm increments. Its bottom surface was embossed to enhance adhesion of the cured material.

### Materials

All chemicals were purchased from Sigma–Aldrich unless otherwise specified and were used without further purification. The soft conductive resin was prepared using acrylamide (AAm, A8887), N,N’‐methylenebis(acrylamide) (MBAA, M7279), potassium chloride (KCl, P3911), and lithium phenyl‐2,4,6‐trimethylbenzoylphosphinate (LAP, 900889). The soft dielectric resin was prepared using 2‐ethylhexyl acrylate (EHA, 290815), ethylene glycol dimethacrylate (EGDMA, 335681), diphenyl(2,4,6‐trimethylbenzoyl)phosphine oxide (TPO, 415952), and aliphatic urethane acrylate oligomer (CN9021, Sartomer). For the stiff structural resin, commercially available urethane‐based photocurable resin (CUKL22, Carima) was used. The luminescent resin was prepared by mixing the stiff structural resin with electroluminescent phosphors (LP‐6861, LP‐6842, and LP‐6813, LWB). For commercially available soft dielectric resins, FLX930 (Stratasys), CYKE05C (Carima), and KER‐4303‐UV (ShinEtsu) were used. For commercially available soft dielectric elastomer, very high bond acrylate tape (VHB, 4905 and 4910, 3 M) was used. Electrical components were connected to the equipment using polyimide‐coated copper wires with a diameter of 0.064 mm (QML‐240 Black, MWS).

### Preparation of Photocurable Resins

To prepare the soft conductive resin, 20 wt.% KCl, 15 wt.% AAm, 0.3 wt.% LAP, and 0.2 wt.% MBAA were added to deionized water. To prepare the soft dielectric resin, a stock solution was prepared by adding CN9021 and EGDMA to EHA. Unless otherwise specified, a stock solution containing 60 wt.% CN9021 and 2 wt.% EGDMA in EHA was used. Subsequently, 2 wt.% TPO was added to the stock solution to complete the preparation of the soft dielectric resin. For the stiff structural resin, unmodified CUKL22 was used. The luminescent resin was prepared by mixing 10 wt.% electroluminescent phosphor into the stiff structural resin immediately before printing.

### Multimaterial 3D Printing

The UV exposure time and intensity were adjusted for each resin during the printing process. For the soft conductive resin, UV light at an intensity of 0.1 W cm^−^^2^ was applied for 8 s per 100 µm layer. For the soft dielectric resin, an intensity of 0.6 W cm^−^^2^ was applied for 5 s per 100 µm layer. For the stiff structural resin, an intensity of 0.6 W cm^−^^2^ was applied for 1.5 s per 50 µm layer. For the luminescent resin, an intensity of 0.6 W cm^−^^2^ was applied for 2 s per 50 µm layer. The UV exposure time was adjusted according to the desired layer thickness.

When multiple layers were printed using the same resin, no additional cleaning steps were performed between layers. However, when transitioning to a different resin for subsequent layers, double cleaning steps were conducted using a sonicator and an air blower to prevent cross‐contamination. The sonication time varied depending on the resin: 5 s for the soft conductive resin, 3 s for the soft dielectric resin, and 10 s for both the stiff structural resin and the luminescent resin. Air blowing was performed in three directions relative to the printing platform: 5s from above, 5 s from the side, and 5 s from below to thoroughly remove IPA and residual resin from the platform.

### Mechanical Tests

To measure Young's modulus, samples were prepared in a rectangular shape with dimensions of 16 mm in length, 5 mm in width, and 1 mm in thickness. For the photocurable resins, the samples were fabricated using the DLPM 3D printer by printing ten 100 µm‐thick layers. For VHB, the samples were cut using a laser cutter. The tests were performed using a universal testing machine (Instron, 3343) at a constant stretch rate of 1 min^−1^.

### Electrical Tests

To measure the dielectric constant and electrical conductivity, 1 mm thick disk‐shaped samples with a diameter of 16 mm were prepared. For the photocurable resins, the samples were fabricated using the DLPM 3D printer by printing ten 100 µm‐thick layers. For VHB, the samples were cut using a laser cutter. The tests were performed using an LCR meter (E4980A, Agilent) equipped with a dielectric test fixture (16451B, Keysight). The samples were placed between two parallel metal plates of the fixture and gently contacted. The dielectric constant and electrical conductivity were obtained through frequency sweeps ranging from 20 Hz to 1 MHz. Measurements at 1 kHz were used as the representative values for the samples.

To measure the dielectric strength, 200 µm thick disk‐shaped samples with a diameter of 16 mm were prepared for the photocurable resins, and 500 µm thick disk‐shaped samples with a diameter of 16 mm were prepared for VHB. For the photocurable resins, the samples were fabricated using the DLPM 3D printer by printing two 100 µm‐thick layers. For VHB, the samples were cut using a laser cutter. The tests were conducted using a waveform generator (33600A, Agilent) connected to a high‐voltage amplifier (30/20A, TREK). The samples were sandwiched between two soft conductive resin electrodes, each shaped as a 1 cm diameter and 5 mm thick disc. The electric field strength applied to the samples was increased at a rate of 1.5 kV/mm s, and the electric field strength at which the leakage current reached 1 mA was recorded.

### Self‐Swelling Tests

To measure the self‐swelling ratio, samples were prepared in a rectangular shape with dimensions of 16 mm in length, 14 mm in width, and 0.4 mm in thickness. The samples were fabricated using the DLPM 3D printer by printing four 100 µm‐thick layers. The samples were submerged in their respective precursor solutions while remaining attached to the printing platform. Weight changes were measured after 5 min and after 24 h. The viscosity of the soft dielectric resin was measured using a viscometer (SV‐10, AND).

### FEM Simulations

Simulations were performed using FEniCS, an open‐source FEM software, based on a previously established finite electro‐elasticity framework.^[^ ^15^ ^]^ Simulation data were analyzed and visualized using ParaView and MATLAB.^[^ ^16^ ^]^ The mDEA was modeled under plane strain conditions and meshed with triangular Taylor‐Hood elements. The soft dielectric resin in the model was subjected to uniform volumetric swelling, and the initial bending angle was measured. With swelling held constant, a 22 kV mm^−1^ electric field was linearly applied, and the horizontal displacement at the actuator tip was recorded. All materials were modeled using a neo‐Hookean material model. In the simulation, Young's moduli measured through mechanical tests were used, with values of 143.5 kPa for the soft dielectric resin, 43.9 kPa for the soft conductive resin, and 283.7 MPa for the stiff structural resin. Similarly, dielectric constant values measured through electrical tests were used, with values of 5.7 for the soft dielectric resin and 5.29 for the stiff structural resin, while the soft conductive resin was assumed to have an infinite dielectric constant.

### Characterization of mDEAs

Unless otherwise specified, the mDEA with a 500 µm‐thick 3D‐patterned active layer was used. The printed mDEA was fixed to a manual linear stage, with its tip aligned with the force sensor (M7‐012, MARK‐10) (Figure S10, Supporting Information). The distance between the mDEA tip and the force sensor was adjusted using the linear stage. An electric field was applied to the mDEA using a waveform generator (33600A, Agilent) connected to a high‐voltage amplifier (30/20A, TREK). Unless otherwise specified, an electric field of 22 kV mm^−1^ was applied for the mDEA with a 500 µm‐thick active layer, while an electric field of 18 kV mm^−1^ was applied for the mDEA with a 300 µm‐thick active layer.

### Characterization of Foot Modules

To measure the resistive force of the foot modules, the microrobot was equipped with the foot modules and placed in their respective environments. The microrobot was then connected to a force sensor (M7‐012, Mark‐10), and the force sensor was pulled using a three‐axis linear stage (SM300SX, Musashi). During the test, the microrobot was pulled at speeds corresponding to its maximum navigation velocity in each environment: 191 cm min^−1^ in the aquatic environment, 52 cm min^−1^ in the smooth environment, 34 cm min^−1^ in the rough environment, and 27 cm min^−1^ in the granular environment (Figure 3E).

### Characterization of Head Modules

Unless otherwise specified, a square‐wave electric field with an amplitude of 15 kV mm^−1^, an offset of 15 kV mm^−1^, and a frequency of 11 kHz was applied to the luminescent modules using a waveform generator (33600A, Agilent) connected to a high‐voltage amplifier (30/20A, TREK). To measure the luminance and electroluminescent spectra of the luminescent modules, a luminance and color meter (CS‐150, Konica Minolta) was used. To detect approaching objects, the proximity module was connected to an external 5 GΩ resistor, and an electrometer (6517A, Keithley) was used to measure the induced voltage across the external load. The resistance change of the illuminometer module was measured using a digital multimeter (34461A, Agilent).

### Microrobot Operation

Navigations in the aquatic, rough, and granular environments were conducted using waveform generators (33600A, Agilent) connected to high‐voltage amplifiers (30/20A, TREK). The active layer thicknesses of the mDEAs composing the main body were 300 µm for the aquatic environment and 500 µm for the rough and granular environments. Unless otherwise specified, during operation in these environments, a sinusoidal electric field with an amplitude of 8 kV mm^−1^ and an offset of 8 kV mm^−1^ was applied, while the frequencies were set to 3, 2, and 4 Hz for the aquatic, rough, and granular environments, respectively.

Navigation in the smooth environment was conducted using two programmable waveform generators (Analog Discovery 2, Digilent) and three high‐voltage amplifiers (10/10B‐HS, 10/10B‐HS, and 30/20A, TREK). One programmable waveform generator was connected to two high‐voltage amplifiers, each connected to a smooth module. The other programmable waveform generator was connected to the remaining high‐voltage amplifier, which was connected to the main body composed of mDEAs with a 500 µm‐thick active layer.

## Conflict of Interest

The authors declare no conflict of interest.

## Author Contributions

W.J.S., Y.‐W.K., and Y.H.L. contributed equally to this work. W.J.S., Y.‐W.K., Y.H.L., and J.‐Y.S. conceived the project and wrote the manuscript. W.J.S. and Y.‐W.K. developed the 3D printing platform and materials. W.J.S. and Y.H.L. designed, conducted, and analyzed the experiments. Y.L. supported the robot design. Y.H.L., S.‐Y.C., and Y.E.C. supported the demonstrations and video recording. J.K. and Y.‐L.P. developed the sequence and software for robot control. B.F.G.A. and X.Z. performed the simulations. X.‐Y.Y. and S.L. reviewed the data and manuscript. J.‐Y.S. and Y.‐L.P. supervised the project.

## Supporting information



Supporting Information

Supplemental Video 1

Supplemental Video 2

Supplemental Video 3

Supplemental Video 4

Supplemental Video 5

Supplemental Video 6

Supplemental Video 7

Supplemental Video 8

## Data Availability

The data that support the findings of this study are available from the corresponding author upon reasonable request.
