# Supplementary material for: All‐3D‐Printed Multi‐Environment Modular Microrobots Powered by Large‐Displacement Dielectric Elastomer Microactuators
Source: Adv Mater. 2025 Sep 9;37(45):e07503. doi: 10.1002/adma.202507503 (PMC12617046; doi:10.1002/adma.202507503)
Supplement: Supplementary file 1 — Supporting Information [file ADMA-37-e07503-s007.pdf]

ADVANCED MATERIALS

Supporting Information

for *Adv. Mater.*, DOI 10.1002/adma.202507503

All-3D-Printed Multi-Environment Modular Microrobots Powered by Large-Displacement Dielectric Elastomer Microactuators

Won Jun Song, Yong-Woo Kang, Yun Hyeok Lee, Junhyung Kim, Bastien F.G. Aymon, Seong-Yu Choi, Yong Eun Cho, Xiao-Yun Yan, Shucong Li, Younghoon Lee, Xuanhe Zhao, Yong-Lae Park and Jeong-Yun Sun**

Supporting Information

All-3D-Printed Multi-Environment Modular Microrobots Powered by Large-Displacement Dielectric Elastomer Microactuators

Won Jun Song†, Yong-Woo Kang†, Yun Hyeok Lee†, Junhyung Kim, Bastien F.G. Aymon, Seong-Yu Choi, Yong Eun Cho, Xiao-Yun Yan, Shucong Li, Younghoon Lee, Xuanhe Zhao, Yong-Lae Park, Jeong-Yun Sun**

SUPPLEMENTAL TEXT

Cross-contamination analysis

In an mDEA, the active layer made of soft dielectric resin is sandwiched between two electrodes composed of soft conductive resin. Although the DLPM 3D printer features a two-step automated cleaning system that utilizes a sonicator and an air blower to minimize cross-contamination between different resins, cross-contamination may still occur during the printing process. When the soft dielectric resin is contaminated by the soft conductive resin, the dielectric strength decreases, rendering it unable to function as an active layer. Consequently, the effective thickness of the active layer becomes thinner than its nominal thickness as cross-contamination occurred at the interfaces with the electrodes. The relationship between the effective thickness and the nominal thickness of the active layer is expressed as follows:

$$t_{\mathrm{eff}} = t_{\mathrm{nom}} - 2t_{\mathrm{cont}} \qquad (S1)$$

where t_{eff} is the effective thickness of the active layer, t_{nom} is the nominal thickness of the active layer, and t_{cont} is the thickness of the contaminated region in the active layer at each interface with electrodes. The thickness of this contaminated region can be determined by analyzing the electric field strength at which electrical breakdown occurs in mDEAs with different nominal active layer thicknesses. The breakdown strength of the soft dielectric resin ($E_{\mathrm{b,SD}}$) can be expressed as

$$E_{\mathrm{b,SD}} = \frac{V_{\mathrm{b,mDEA}}}{t_{\mathrm{eff}}} = \frac{E_{\mathrm{b,mDEA}} \cdot t_{\mathrm{nom}}}{t_{\mathrm{nom}} - 2t_{\mathrm{cont}}} \qquad (S2)$$

where $V_{\mathrm{b,mDEA}}$ is the voltage and $E_{\mathrm{b,mDEA}}$ is the electric field strength at which electrical breakdown occurs in the mDEA. In an mDEA with a nominal active layer thickness of 500

µm, electrical breakdown occurred at an electric field strength of 22 kV/mm, while in an mDEA with a nominal active layer thickness of 300 µm, it occurred at 18 kV/mm (Figure 2G). Substituting these values into Equation S2:

$$E_{\mathrm{b,SD}} = \frac{22\ \mathrm{kV/mm} \cdot 0.5\ \mathrm{mm}}{0.5\ \mathrm{mm} - 2t_{\mathrm{cont}}} = \frac{18\ \mathrm{kV/mm} \cdot 0.3\ \mathrm{mm}}{0.3\ \mathrm{mm} - 2t_{\mathrm{cont}}} \qquad \text{(S3)}$$

The thickness of the contaminated region is calculated to be 53.57 µm. Consequently, the effective active layer thicknesses were determined to be 392.86 µm for the mDEA with a nominal active layer thickness of 500 µm and 192.86 µm for the mDEA with a nominal active layer thickness of 300 µm.

Voltage Sequence for Smooth Environment Navigation

The microrobot was able to walk forward or backward in a smooth environment by precisely controlling the voltage sequences applied to its two smooth modules and the main body. The forward walking sequence was divided into six steps (Figure S16A, Supporting Information):

1. Voltage was applied to the front foot to induce electrostatic adhesion with the ground.
2. Voltage was applied to the main body to induce contraction.
3. Reverse voltage was applied to the front foot to eliminate electrostatic adhesion with the ground.
4. Voltage was applied to the rear foot to induce electrostatic adhesion with the ground.
5. The voltage applied to the main body was removed to induce extension.
6. Reverse voltage was applied to the rear foot to eliminate electrostatic adhesion with the ground.

The backward walking sequence follows the similar six-step process in reverse order (Figure S16B, Supporting Information):

1. Voltage was applied to the rear foot to induce electrostatic adhesion with the ground.
2. Voltage was applied to the main body to induce contraction.
3. Reverse voltage was applied to the rear foot to eliminate electrostatic adhesion with the ground.
4. Voltage was applied to the front foot to induce electrostatic adhesion with the ground.
5. The voltage applied to the main body was removed to induce extension.
6. Reverse voltage was applied to the front foot to eliminate electrostatic adhesion with the ground.

The smooth modules adjusted friction with the ground through electrostatic adhesion. When voltage was applied to the smooth module, a strong electric field formed between the electrodes, polarizing the surface beneath it and generating electrostatic adhesion between the smooth module and the ground. However, this adhesion did not disappear immediately when the voltage was removed and persisted for a short duration. To quickly eliminate this residual electrostatic adhesion, a reverse voltage was applied to resolve the issue effectively.

SUPPLEMENTAL FIGURE

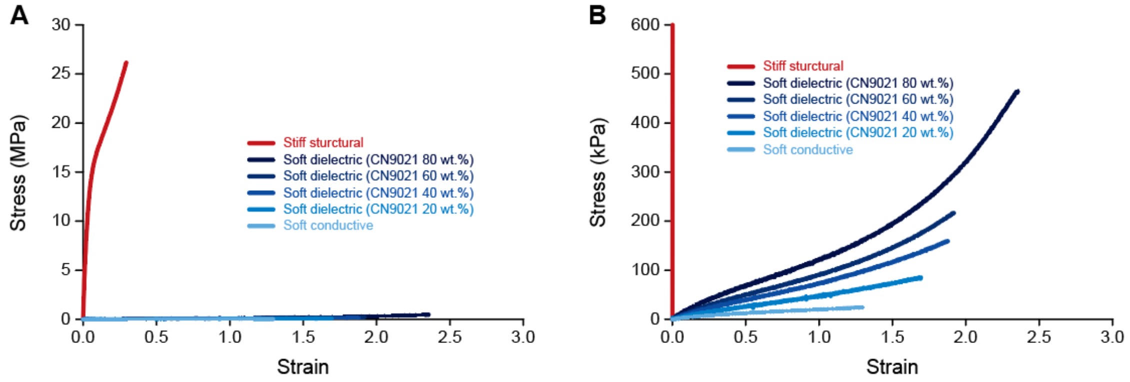

Figure S1. Mechanical properties of photocurable resins. A) Stress-strain curves of stiff structural, soft dielectric, and soft conductive resins, stretched until rupture. B) Enlarged view of the low-stress region to highlight differences among the soft resins.

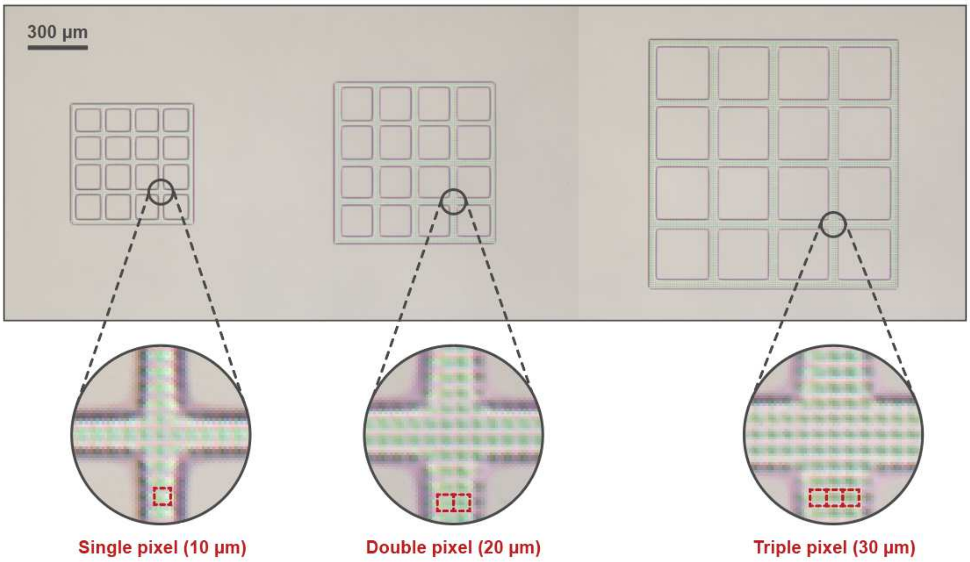

Figure S2. Resolution of the DLPM 3D printer. Grid structures were printed using stiff structural resin to assess the printer's resolution. The DLPM 3D printer has a pixel size of 10 μm × 10 μm**,** suitable for mesoscale manufacturing.

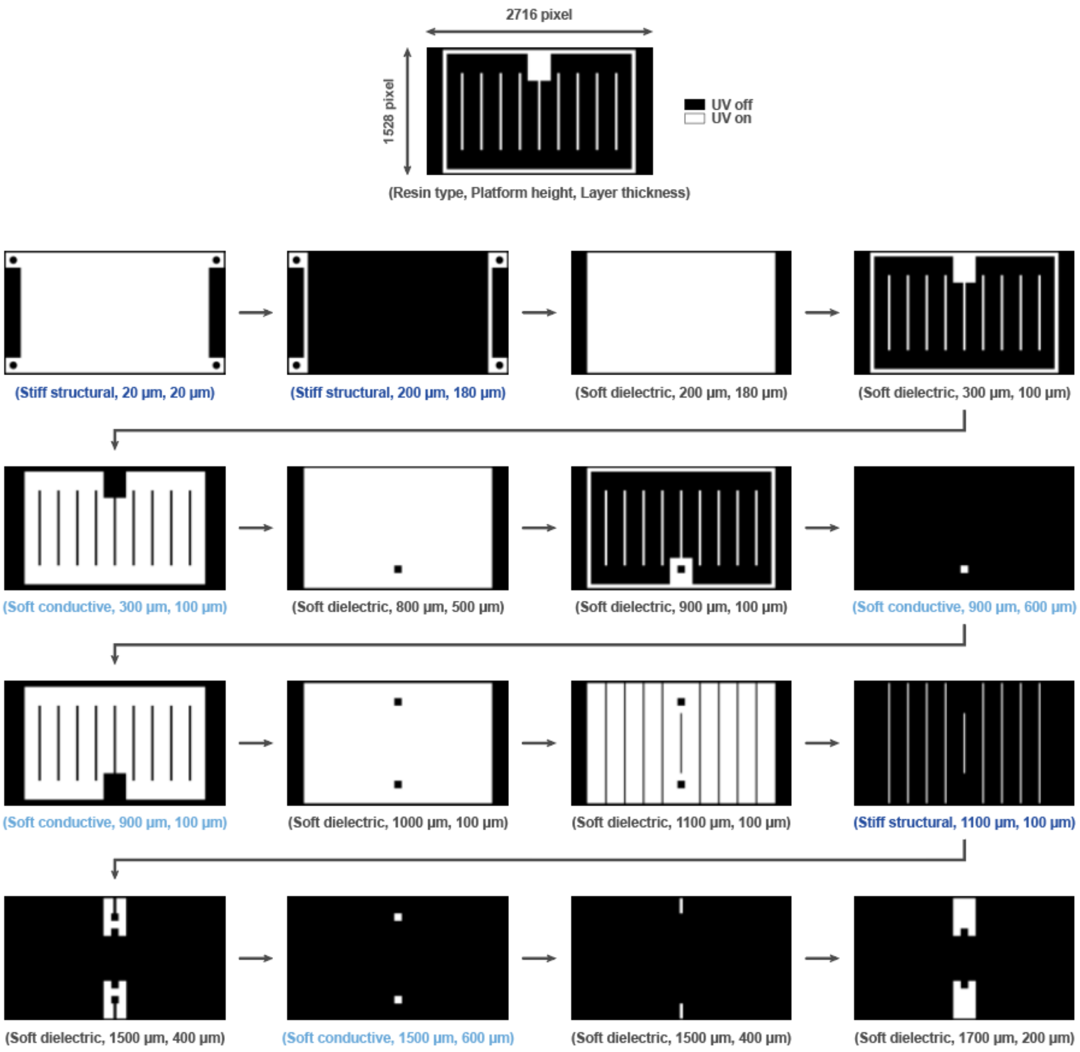

Figure 3. Printing sequence of the main body. The main body was fabricated using 16 layers of three different resins. In the image for each layer, white regions represent UV-exposed areas, while black regions indicate non-UV-exposed areas. The annotations below each image specify, from left to right, the type of resin used, the platform height relative to the bottom of the resin chamber, and the thickness of the printed layer.

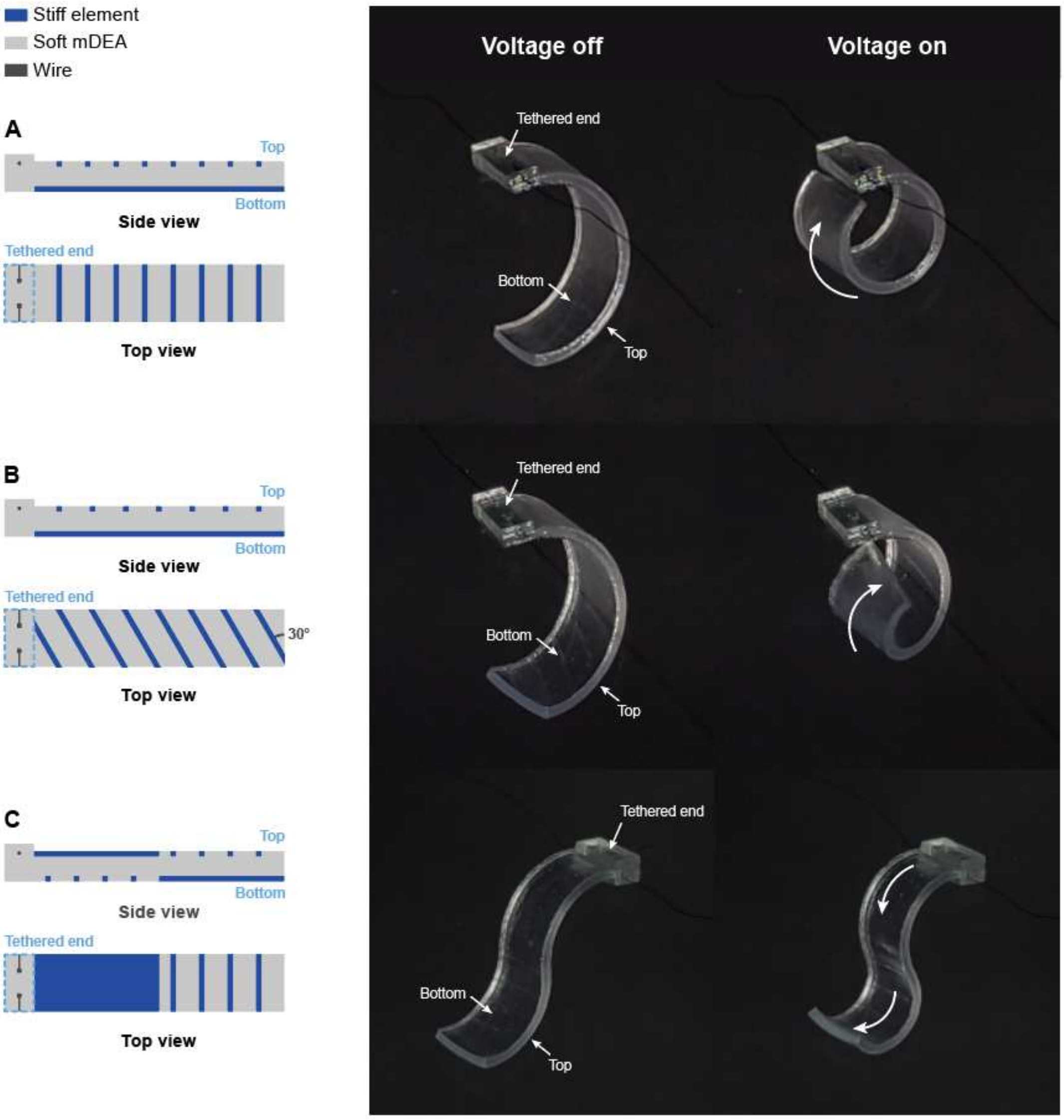

Figure S4. Control of mDEA bending directions using stiff elements. The bending behavior of a mDEA can be controlled by the arrangement of stiff elements. A) A standard-bending mDEA is fabricated by placing a stiff film at the bottom and stiff fibers parallel to the actuator at the top. B) A helical-bending mDEA is fabricated by placing a stiff film at the bottom and stiff fibers at a 30-degree angle to the actuator at the top. C) A dual-bending mDEA is fabricated by reversing the arrangement of the stiff film and fibers relative to the actuator's center. An electric field strength of 16 kV/mm was applied in each demonstration.

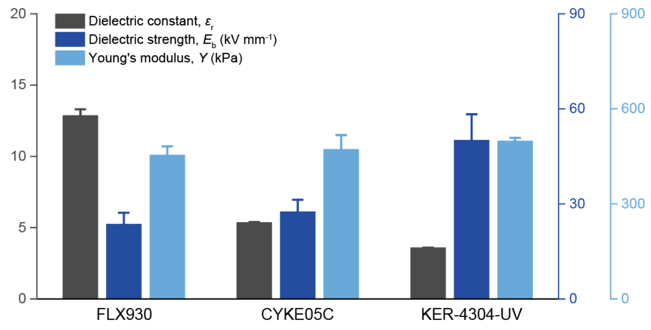

Figure S5. Electromechanical properties of conventional soft dielectric resins for DLP 3D printing applications. From left to right, the graph shows the dielectric constant, and dielectric strength and Young's modulus of soft dielectric resins. Error bars indicate SDs; N=3.

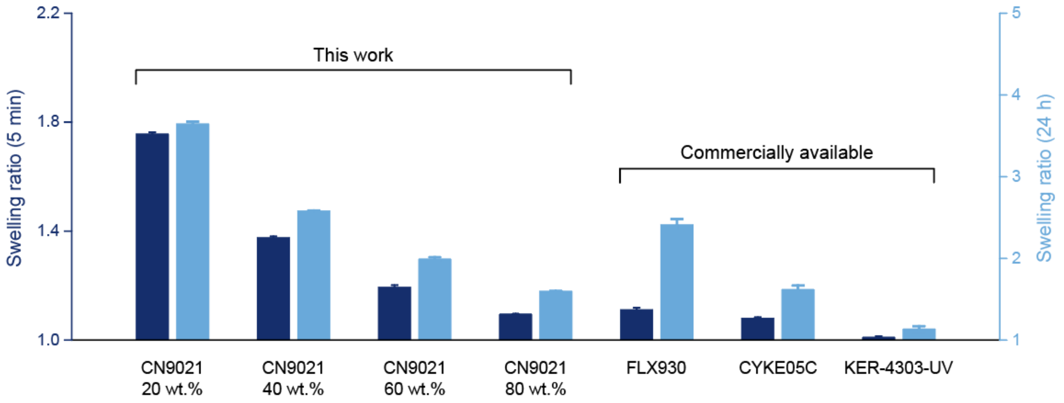

120 **Figure S6.** Self-swelling behavior of the soft resins. Soft resins with a low Young's modulus in the kilopascal range undergo self-swelling during the printing process. The swelling ratio of a cured polymer was measured after 5 minutes and 24 hours of being submerged in its precursor solution at room temperature. Error bars indicate SDs; N=3.

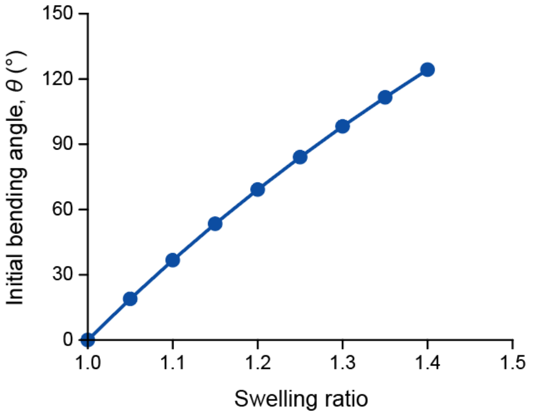

Figure S7. FEM simulation of the initial bending angle as a function of the swelling ratio. Finite element simulations were performed using the FEniCS software[1] to investigate whether the self-swelling of the soft dielectric resin over a few minutes (Figure S6)—the time typically required to print each layer—could induce sufficient initial bending. In the simulation, the initial bending angle was generated by uniform volumetric swelling of the soft dielectric resin in a model in flat configuration.

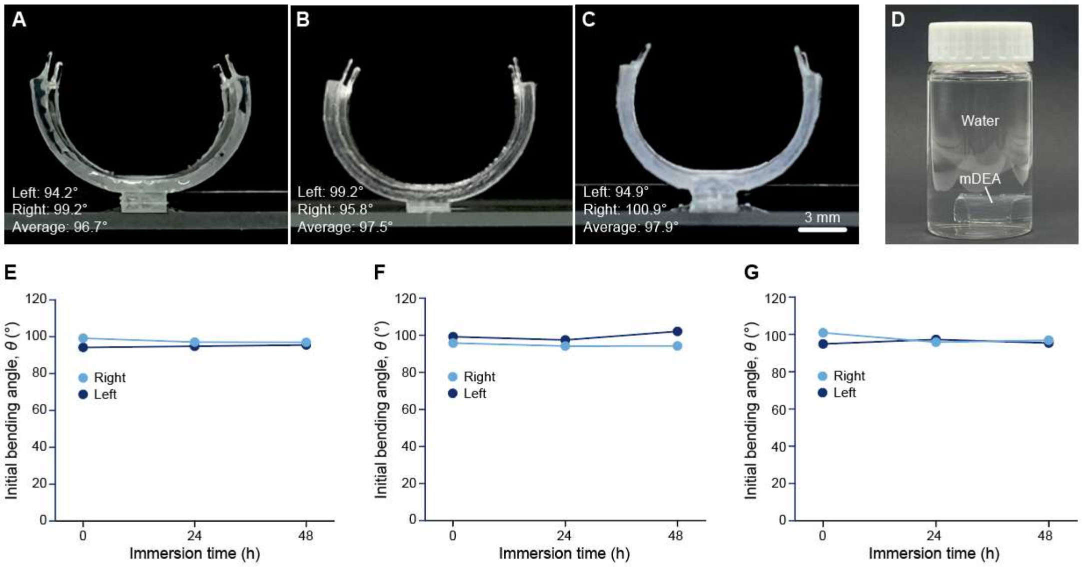

Figure S8. Reproducibility and stability of the initial bending angle. A–C) To evaluate the reproducibility of the initial bending angle, three mDEAs were printed on different days using a soft dielectric resin composed of CN9021 60 wt.% and EGDMA 2 wt.%. The initial bending angles of the mDEAs consistently reached values close to the optimal angle of 98.2°. Scale bar, 3 mm. D) The stability of the initial bending angle in a humid environment was tested by immersing the mDEAs in water. E–G) The initial bending angles of the three mDEAs were measured as a function of immersion time in water. The mDEAs maintained stable initial bending angles for 48 hours.

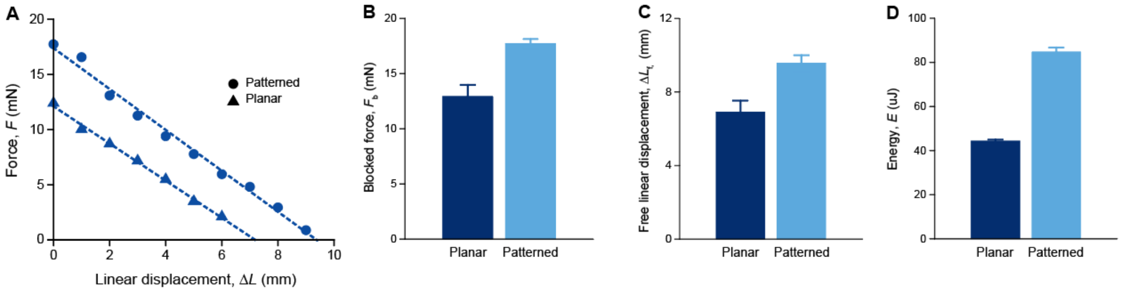

Figure S9. Effect of active layer patterns on actuation performance. A) Relationship between force and linear displacement for mDEAs with planar and 3D-patterned active layers. The 3D patterns enhance force transfer between the active layer and stiff elements during actuation, resulting in increased force and linear displacement. B-D) (B) Blocked force, (C) free linear displacement, and (D) energy output of mDEAs with planar and 3D-patterned active layers. Through the 3D patterns, the blocked force, free linear displacement, and energy output of the mDEA increased by 37.1%, 38.5%, and 91.0%, respectively. Error bars indicate SDs; N=3.

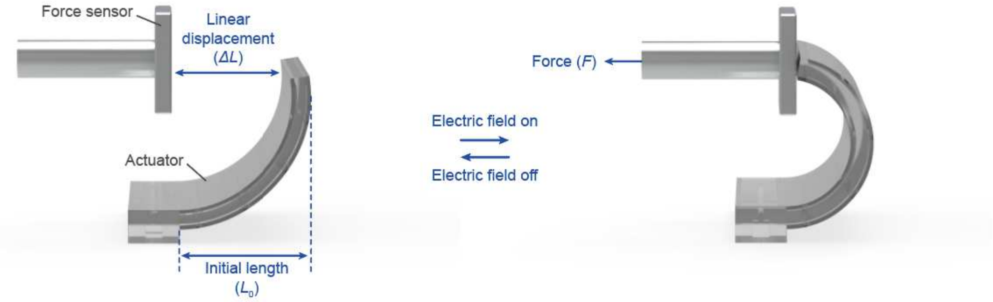

Figure S10. Actuation performance measurement setup. The actuation performance of the mDEA was measured using a force sensor. The distance between the force sensor and the actuator in the resting state corresponds to the linear displacement (ΔL), while the force measured by the sensor upon applying electric field is the actuation force (F). The force at zero linear displacement is defined as the blocked force (F_b), and the linear displacement at which the actuation force measured by the sensor reaches zero is defined as the free linear displacement (ΔL_f). The length of the actuator in the resting state was defined as the initial length (L_0).

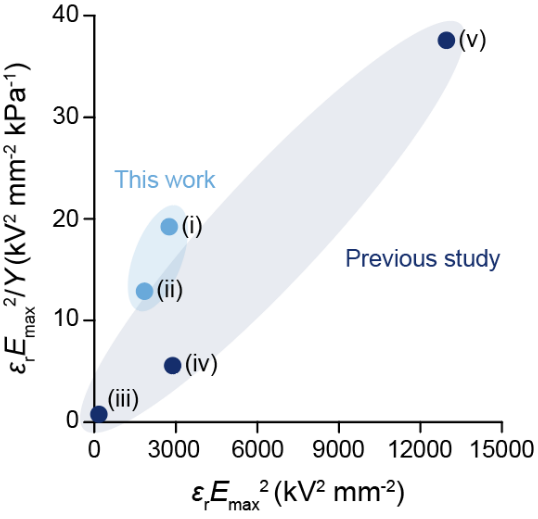

Figure S11. Ashby plot comparing the electromechanical properties of soft dielectric materials at the device level. Due to cross-contamination during the DLPM 3D printing process, the maximum electric field applicable to the printed mDEAs (Figure 2K) is lower than the dielectric strength of the developed soft dielectric resin (Figure 2B). To reflect the influence of the fabrication process, the maximum stress (proportional to $\varepsilon_r E_{max}{}^2$) and maximum strain (proportional to $\varepsilon_r E_{max}{}^2/Y$) of the developed soft dielectric resin were recalculated using the maximum electric field applicable to the device (E_{max}) instead of the dielectric strength of the soft dielectric materials (E_b) and compared with those of soft dielectric materials used in the previously reported soft electrical devices. The comparison includes soft dielectric materials used in (i) the developed mDEA with a 500 µm-thick active layer, (ii) the developed mDEA with a 300 µm-thick active layer, (iii) the flexible electroluminescent device reported in Reference 2, (iv) the DEA reported in Reference 3, and (v) the DEA reported in Reference 4.

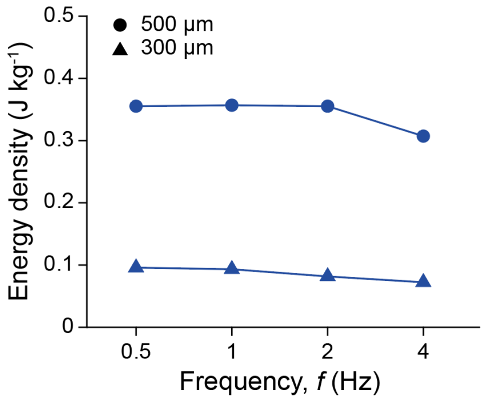

Figure S12. Effect of operating frequency on energy density. Energy density was calculated from the relationship between force and linear displacement (Figure 2M). The energy density showed a slight decrease with increasing operating frequency.

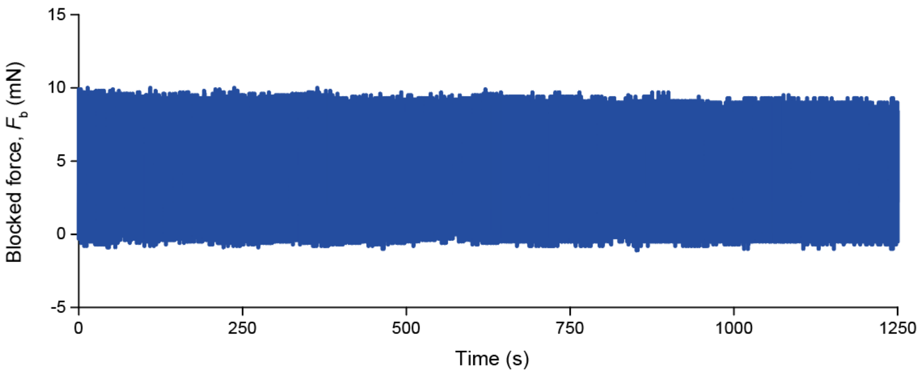

Figure S13. Cyclic test of the mDEA. The mDEA demonstrated stable performance over 5000 cycles at a frequency of 4 Hz for a total of 1250 seconds. The electric field strength applied during the test was 16 kV/mm.

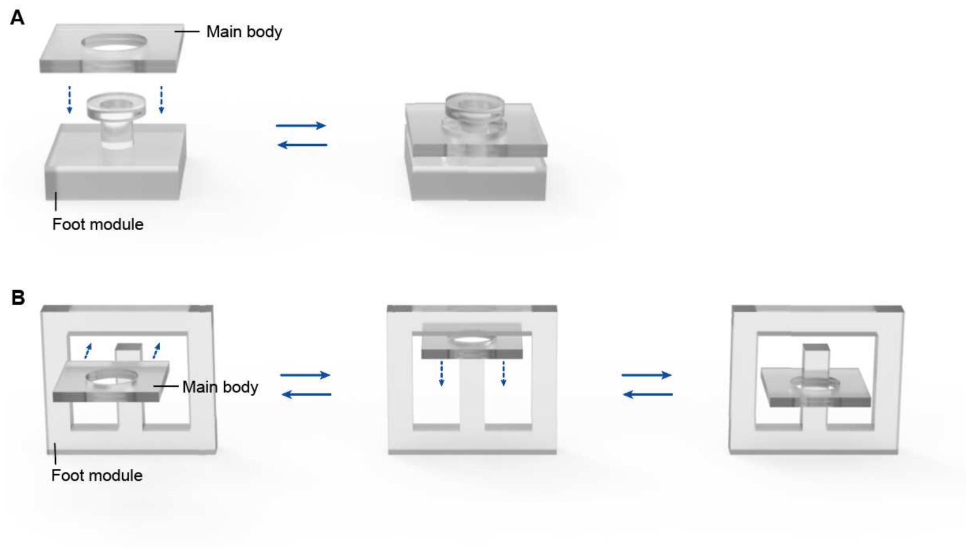

Figure S14. Two assembly methods for the foot module. A) Illustration of the rigid coupling method. This method is used to connect the main body to the smooth modules, providing a secure connection without any play. B) Illustration of the loose coupling method. This method is used to connect the main body to the aquatic or rough/granular modules, allowing the foot modules to freely adjust their angle according to the surrounding environment while remaining connected to the main body.

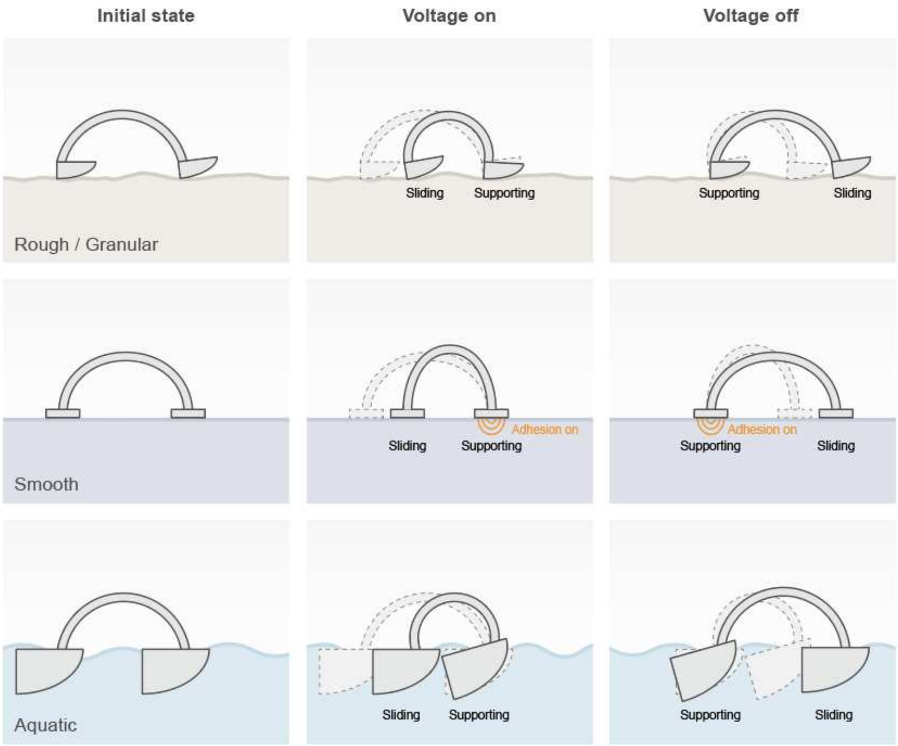

195 **Figure S15.** Walking mechanism of the microrobot. Each foot module is designed to generate a greater resistive force during the supporting phase than during the sliding phase. This enables the microrobot to move forward by transitioning the phases between the front and rear foot.

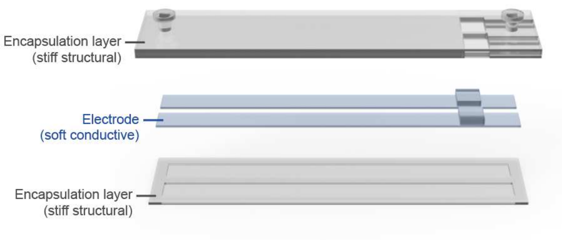

Figure S16. Exploded view of the smooth module. The smooth module adopts an interdigitated electrode design. When a voltage is applied to the smooth module, a strong electric field is formed between the electrodes, polarizing the surface beneath it and generating electrostatic adhesion between the smooth module and the ground[5].

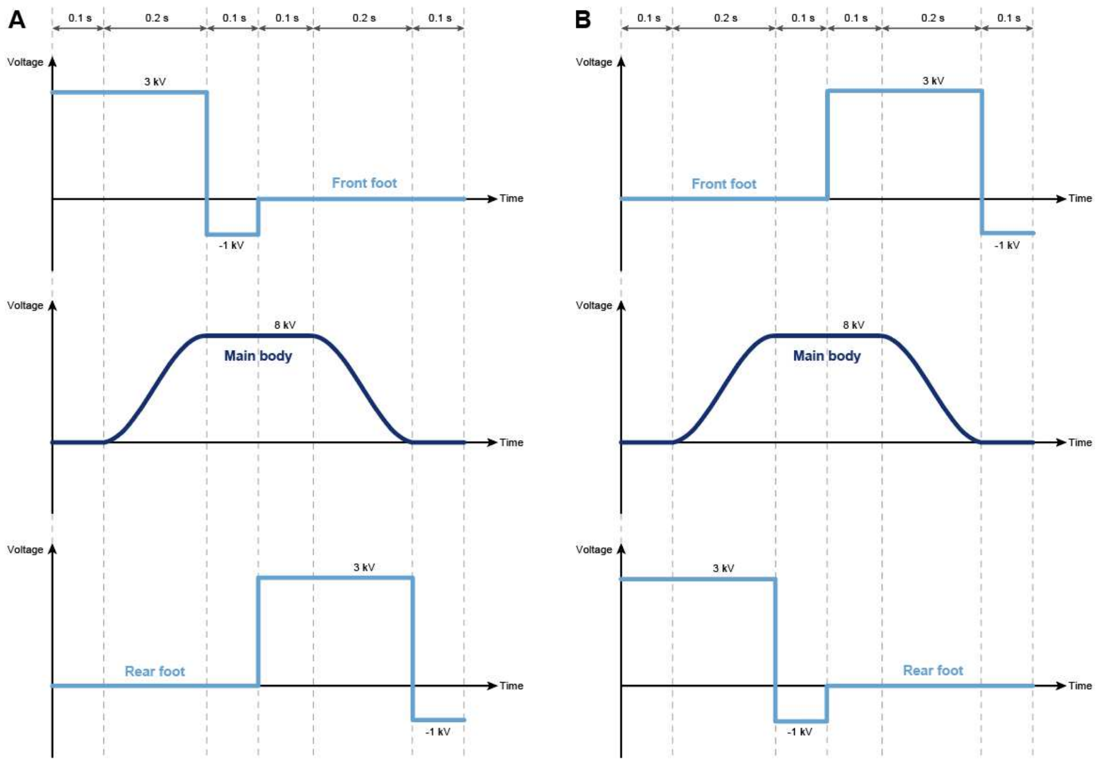

Figure S17. Voltage sequence for smooth environment navigation. A, B) Voltage sequences that enable the microrobot to walk (A) forward and (B) backward in the smooth environment. A voltage of 3 kV is applied to the smooth modules to induce electrostatic adhesion with the ground, while a reverse voltage of 1 kV is applied to immediately eliminate the adhesion. By applying a reverse voltage for a short duration, the accumulated charges on the electrodes are removed, enabling immediate detachment. Each cycle consists of six steps and requires a total of 0.8 seconds.

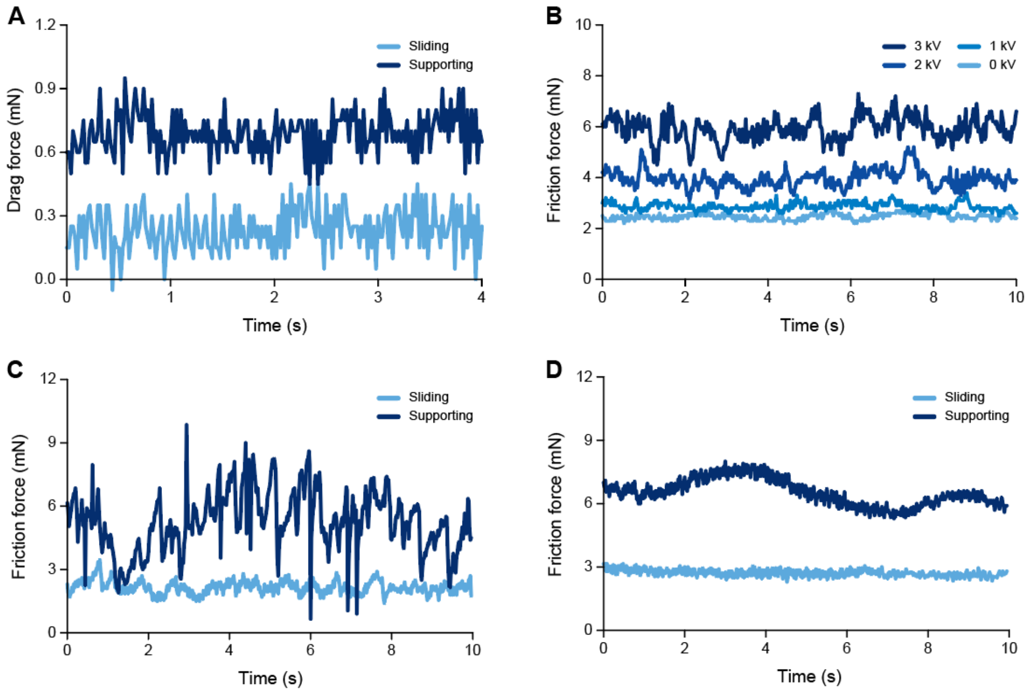

Figure S18. Resistive force measurements of the foot modules. A) Drag force of the aquatic module during the sliding and supporting phases. B) Friction force of the smooth module at various applied voltage. As the applied voltage increases, the electrostatic adhesion with the surface increases, resulting in a higher friction force. C, D) Friction force of the rough/granular module in (C) rough and (D) granular environments during the sliding and supporting phases.

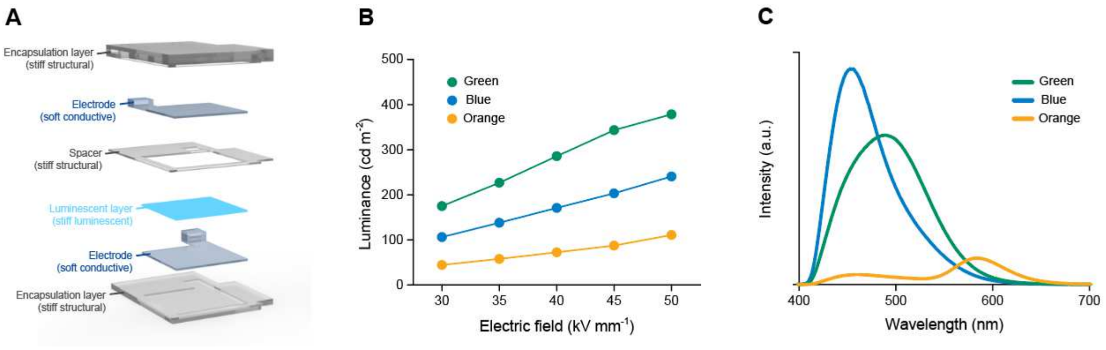

Figure S19. Characterization of the luminescent module. A) Exploded view of the luminescent module. When an AC electric field is applied to the electrodes, light is emitted from the electroluminescent layer containing electroluminescent phosphors via excitations within intrinsic heterojunctions. B) Luminance as a function of the applied electric field strength. The luminescent module emits brighter light as the applied electric field strength increases. C) Electroluminescence spectra of different types of electroluminescent phosphors. The color of the luminescent module depends on the electroluminescent phosphors used in the luminescent layer[2, 6].

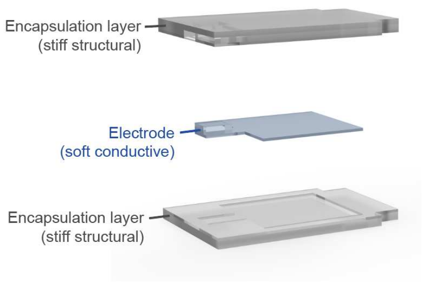

Figure S20. Exploded view of the proximity module. The object usually has static charges on its surface since contact with its surroundings charges the object through contact electrification. When the charged object approaches the proximity module, the electric fields originating from the object induce a voltage in the electrode of the proximity module. The magnitude of the induced voltage can be used to estimate the relative distance to the object but does not indicate its direction. Determining the direction would require an array of proximity modules, with voltage comparisons between individual modules enabling the detection of both the relative distance and the direction of the object[5c, 7].

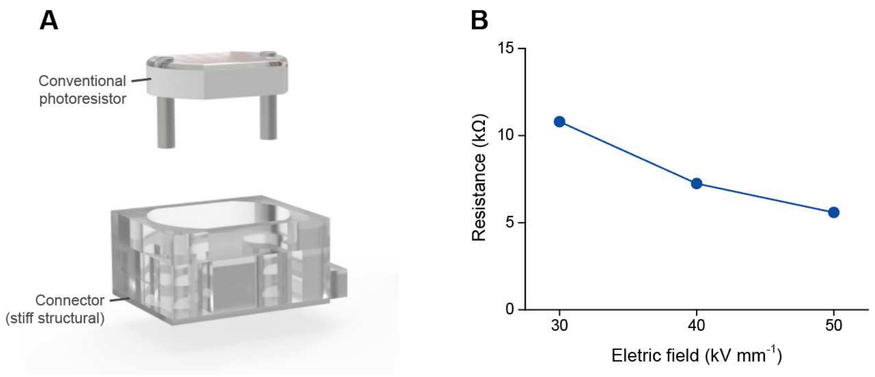

Figure S21. Characterization of the illuminometer module. A) Exploded view of the illuminometer module. The illuminometer module was developed by integrating a cadmium sulfide-based commercial photoresistor with a 3D-printed connector. B) Resistance changes of the illuminometer module as a function of the electric field strength applied to the luminescent module. As the luminescent module emits stronger light, the resistance of the illuminometer module decreases. The distance between the two modules was fixed at 1 cm.

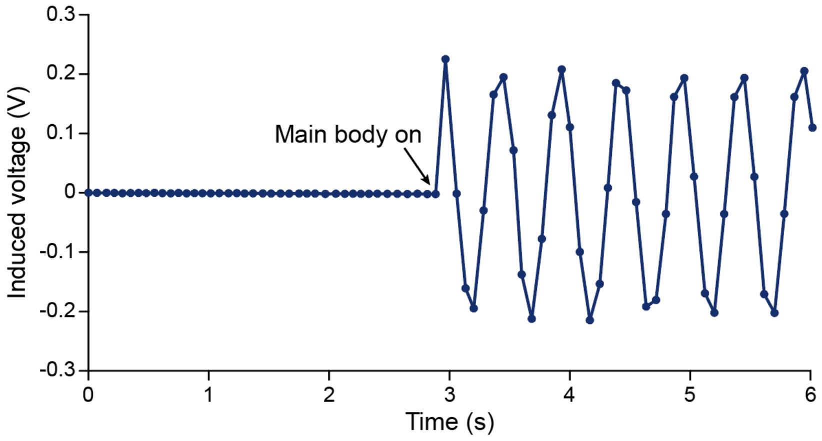

Figure S22. Signal interference between the main body and the proximity module. A strong electric field is required for the main body to generate mechanical movement, which can interfere with the proximity module assembled at the central interlocking interface. To avoid the interference, the proximity module is active only when the microrobot is stationary. Once a human is detected, the proximity module triggers the main body to walk forward by the set distance and then turns off during the movement. After avoiding the collision, the proximity module is reactivated to resume detecting nearby humans.

SUPPLEMENTAL TABLE

Environment	Mass (g)	Maximum velocity (cm s^{-1})	Input power (mW)	Cost of transport
Aquatic	1.10	3.19	9.85	28.68
Smooth	0.63	0.88	17.35	321.46
Rough	0.84	0.58	15.14	318.40
Granular	0.84	0.46	25.59	678.46

Table S1. Cost of transport of the microrobot in various environments. Cost of transport is a dimensionless metric used to compare the energy efficiency of moving robots and is defined as (input power) / (mass × gravity × velocity)[8]. The cost of transport of the microrobot assembled with different foot modules was calculated in aquatic, smooth, rough, and granular environments.

SUPPLEMENTAL VIDEO

Video S1. Fabrication of luminescent modules using the DLPM 3D printer. Eight luminescent modules were fabricated simultaneously in just 30 minutes using the DLPM 3D printer. Each module comprises 27 layers made of soft conductive, stiff structural, and luminescent resins. This highlights the potential of DLPM 3D printing technology for the mass production of mesoscale components for microrobots.

Video S2. Operation of the main body. Powered by two mDEAs capable of generating large linear displacements, the main body provides substantial mechanical movement to overcome obstacles on land and rapidly propel the robot on water. During operation, an electric field of 16 kV/mm was applied, with a frequency sweep ranging from 0.5 Hz to 4 Hz.

Video S3. Control of mDEA bending directions using stiff elements. The precise arrangement of stiff elements enabled the development of mDEAs exhibiting diverse bending modes. By adjusting the position and orientation of stiff films and fibers, we fabricated mDEAs capable of standard-bending, helical-bending, and dual-bending. During operation, an electric field of 16 kV/mm was applied, with a frequency sweep ranging from 0.5 Hz to 4 Hz.

Video S4. Demonstrations of multi-environment navigation. The modular microrobot seamlessly adapts to four distinct environments and navigates them effectively by simply replacing its foot modules. The microrobot achieves walking velocities of up to 53.2 BL/min in aquatic environments, 21.8 BL/min in smooth environments, 17.1 BL/min in rough environments, and 13.4 BL/min in granular environments.

Video S5. Demonstration of real-time robot-robot interaction. Two modular microrobots, a leader robot and a follower robot, interacted in real time using optical signals. The leader robot, equipped with a luminescent module, used optical cues to signal the follower robot, which was equipped with an illuminometer module, indicating when to move. The leader robot successfully controlled the follower robot’s movements in real time, even at a distance of several centimeters.

Video S6. Demonstration of real-time human-robot interaction. The modular microrobot, equipped with a proximity module, can detect approaching objects in real time. The robot was programmed to automatically move forward a set distance to avoid collisions whenever a human approached, successfully demonstrating real-time collision avoidance in multiple times.

Video S7. Series connection for increased speed. Two modular microrobots were connected in series using a series-connecting module. The series-connected robot, with its extended step length, accelerated faster and achieved a maximum velocity 50% greater than the single-unit robot. This demonstrated that collaboration effectively overcomes speed limitations.

Video S8. Parallel connection for increased force output and control. Two modular microrobots were connected in parallel using a parallel-connecting module. When both units operated simultaneously, the parallel-connected robot exhibited a stronger force output compared to a single-unit robot. In a demonstration pulling a 110 g rubber duck, which is equivalent to 100 times the weight of the 1.1 g single-unit robot, the parallel-connected robot achieved a maximum velocity 90% faster than the single-unit robot. Activating only one of the two parallel-connected units enabled precise directional control, allowing the robot to complete a 180° turn in just 7.9 seconds with the stationary unit serving as a pivot. During this process, the robot's rotational speed gradually increased and reached a maximum angular velocity of 28.7°/s.

REFERENCES

[1] a)M. Alnæs, J. Blechta, J. Hake, A. Johansson, B. Kehlet, A. Logg, C. Richardson, J. Ring, M. E. Rognes, G. N. Wells, *Archive of numerical software* **2015**, 3; b)A. Logg, K.-A. Mardal, G. Wells, *Automated solution of differential equations by the finite element method: The FEniCS book*, Vol. 84, Springer Science & Business Media, **2012**.

[2] P. Zhang, I. M. Lei, G. Chen, J. Lin, X. Chen, J. Zhang, C. Cai, X. Liang, J. Liu, *Nature Communications* **2022**, 13, 4775.

[3] N. M. Larson, J. Mueller, A. Chortos, Z. S. Davidson, D. R. Clarke, J. A. Lewis, *Nature* **2023**, 613, 682.

[4] A. Chortos, J. Mao, J. Mueller, E. Hajiesmaili, J. A. Lewis, D. R. Clarke, *Advanced Functional Materials* **2021**, 31, 2010643.

[5] a)S. D. De Rivaz, B. Goldberg, N. Doshi, K. Jayaram, J. Zhou, R. J. Wood, *Science Robotics* **2018**, 3, eaau3038; b)G. Gu, J. Zou, R. Zhao, X. Zhao, X. Zhu, *Science Robotics* **2018**, 3, eaat2874; c)Y. Lee, W. J. Song, Y. Jung, H. Yoo, M.-Y. Kim, H.-Y. Kim, J.-Y. Sun, *Science robotics* **2020**, 5, eaaz5405.

[6] C. Larson, B. Peele, S. Li, S. Robinson, M. Totaro, L. Beccai, B. Mazzolai, R. Shepherd, *science* **2016**, 351, 1071.

[7] W. J. Song, Y. Lee, Y. Jung, Y.-W. Kang, J. Kim, J.-M. Park, Y.-L. Park, H.-Y. Kim, J.-Y. Sun, *Science advances* **2021**, 7, eabg9203.

[8] a)R. Baines, S. K. Patiballa, J. Booth, L. Ramirez, T. Sipple, A. Garcia, F. Fish, R. Kramer-Bottiglio, *Nature* **2022**, 610, 283; b)C. A. Aubin, R. H. Heisser, O. Peretz, J. Timko, J. Lo, E. F. Helbling, S. Sobhani, A. D. Gat, R. F. Shepherd, *Science* **2023**, 381, 1212.
